# Glacial isostatic adjustment directed incision of the Channeled Scabland by Ice Age megafloods

**DOI:** 10.1073/pnas.2109502119

**Published:** 2022-02-14

**Authors:** Tamara Pico, Scott R. David, Isaac J. Larsen, Alan C. Mix, Karin Lehnigk, Michael P. Lamb

**Affiliations:** ^a^Division of Geological and Planetary Sciences, California Institute of Technology, Pasadena, CA 91125;; ^b^Department of Geosciences, University of Massachusetts Amherst, Amherst, MA 01003;; ^c^Department of Watershed Sciences, Utah State University, Logan, UT 84322;; ^d^College of Earth, Ocean, and Atmospheric Sciences, Oregon State University, Corvallis, OR 97331

**Keywords:** Channeled Scabland, glacial isostatic adjustment, erosional history

## Abstract

During the last deglaciation, dozens of glacial outburst floods—among the largest known floods on Earth—scoured the Channeled Scabland landscape of eastern Washington. Over this same period, deformation of the Earth’s crust in response to the growth and decay of ice sheets changed the topography by hundreds of meters. Here, we investigated whether glacial isostatic adjustment affected routing of the Missoula floods and incision of the Channeled Scabland from an impounded, glacial Lake Columbia. We used modern topography corrected for glacial isostatic adjustment as an input to flood models that solved the depth-averaged, shallow water equations and compared the results to erosion constraints. Results showed that floods could have traversed and eroded parts of two major tracts of the Channeled Scabland—Telford-Crab Creek and Cheney–Palouse—near 18 ka, whereas glacial isostatic adjustment limited flow into the Cheney–Palouse tract at 15.5 ka. Partitioning of flow between tracts was governed by tilting of the landscape, which affected the filling and overspill of glacial Lake Columbia directly upstream of the tracts. These results highlight the impact of glacial isostatic adjustment on megaflood routing and landscape evolution.

## Significance

The glacial Lake Missoula outburst floods are among the largest known floods on Earth. Dozens of these floods scoured the landscapes of eastern Washington during the last Ice Age, from 18 to 15.5 thousand years ago, forming what is known as the Channeled Scabland. We explored how changes in topography due to the solid Earth’s response to ice sheet loading and unloading influenced the history of megaflood routing over the Channeled Scabland. We found that deformation of Earth’s crust played an important role in directing the erosion of the Channeled Scabland.

On glacial timescales, the growth and decay of continental ice sheets deform the solid Earth, producing subsidence under ice-loaded regions and inducing uplift in the periphery of the ice sheet (1). Rates of Ice Age crustal deformation are sufficient to influence landscape dynamics, including river erosion and drainage basin reorganization (2, 3). The course of ancient, glacial outburst floods was likely influenced by glacial isostatic adjustment (GIA), and reconstructing these events informs our understanding of how floods shape landscapes on Earth and Mars (4). However, paleomegaflood reconstructions often use the present-day topography to approximate past landscapes (5–8). The potential influence of GIA on flood routing has been recognized (9, 10) but remains largely unexplored.

Here, we focused on the Channeled Scabland, where dozens of Pleistocene megafloods sourced from an ice-dammed glacial Lake Missoula scoured the landscapes of Idaho and eastern Washington (4, 9, 11–17). The landscape includes many deeply carved canyons and tracts of land with scoured basalt and stream-lined loess deposits, indicating floods traversed and eroded several different pathways at different times (16–19). GIA caused crustal deformation in the Channeled Scabland with rates up to 10 mm/y (2) orders of magnitude above regional tectonic uplift rates (20–22) and, therefore, may have influenced flood routing.

Although a century of study has improved our understanding of the Channeled Scabland, the magnitude and timing of discharge in different flood pathways remains an active area of research (9, 18, 23, 24), such that any model of flooding requires assumptions. We used relatively simple, yet plausible, numerical experiments to test whether GIA could have had a substantial impact on flood routing and erosion for two major scabland tracts, Cheney–Palouse and Telford-Crab Creek. To this end, we modeled GIA to reconstruct the topography of the Channeled Scabland at different times during the period of Ice Age flooding. We neglected ice–dam break dynamics, three-dimensional (3D) hydrodynamics, variable hydraulic roughness, and sediment transport and erosion. We did not model landscape evolution due to erosion. We explored a limited set of possible flood durations, magnitudes, and hydrograph shapes. We held all model inputs (e.g., flood size, duration, and lake level) constant between two simulation sets to isolate the role of GIA on flood routing. We tested the plausibility of the modeled hydrographs by comparing it to thresholds for bedrock incision.

## Channeled Scabland Tracts and Modeling Approach

**Fig. 1.**
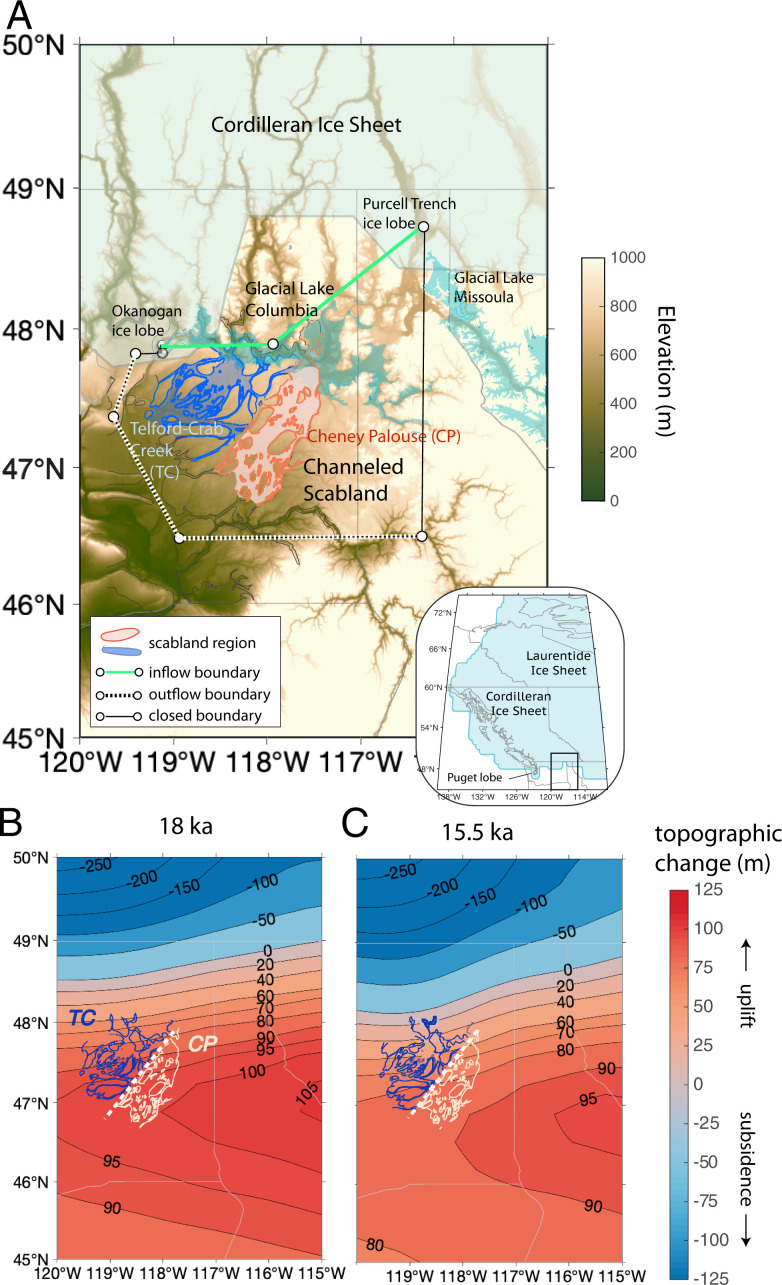
(*A*) Present-day elevation of Channeled Scabland region. Outlined regions are the Telford-Crab Creek (blue) and Cheney–Palouse (orange) scabland tracts, which contain basalt-eroded regions (35). Glacial Lake Columbia and glacial Lake Missoula shown in shaded turquoise. Shaded light blue regions denote the maximum Cordilleran ice sheet extent used in ice history GI31-ANUed-PC2 (resolution = 78 km; *Methods*). White circles and black lines mark the model domain used for flood simulations, dashed white lines show open (outflow) boundaries, and green lines show the location of the inlet (inflow boundaries) where simulated discharge spilled from glacial Lake Columbia onto the Columbia Plateau. Topographic change at 18 (*B*) and 15.5 ka (*C*) due to GIA using ice history GI31-ANUed-PC2. Blue regions were subsided (lower elevation) relative to present-day topography or equivalently modern global mean sea surface elevation, whereas red regions were uplifted (higher elevation) relative to present-day topography. Light gray lines in *A* and *B* show US state borders.

Downstream of glacial Lake Missoula, the Columbia River was dammed by the advancing Okanogan ice lobe (25, 26), forming glacial Lake Columbia between ∼18 to 15.5 ka (18). Evidence of flood deposits (12, 27) suggests that the Missoula floods were responsible for spillover from glacial Lake Columbia into two major channel systems in the eastern part of the Channeled Scabland: the Cheney–Palouse and the Telford-Crab Creek tracts (17, 19) (Fig. 1*A*). These regions consist of zones of flood-scoured basalt (scabland regions; Fig. 1*A* and *Methods*) and neighboring, loess-covered terrain (non-scabland regions). We focused on the Telford-Crab Creek and Cheney–Palouse tracts and did not consider flood pathways into Grand Coulee, Moses Coulee, or the Columbia Valley.

To model topographic change due to ice sheet loading, we used a gravitationally self-consistent GIA model with deglacial ice history GI31-ANUed-PC2, which incorporates recent constraints on the Cordilleran ice sheet–melting history (28, 29), including regional ice lobe margins (*Methods*). The topography also was affected by erosion of loess and the underlying basaltic bedrock during floods (17, 24, 30). However, to isolate the effect of GIA on flooding, and because the evolution of topographic change due to incision is unknown, we followed most studies (5, 6, 10, 16, 17, 31, 32) and used the present-day topography, correcting only for GIA.

To simulate floods we used ANUGA, a two-dimensional, hydrodynamic model (33), to solve the depth-averaged, shallow water equations for spillover of a single flood entering glacial Lake Columbia. Since we were interested in the role of GIA on the formation of the Channeled Scabland, we focused on the period when glacial Lake Columbia existed, from 18 to 15.5 ka (*SI Appendix*, section 1). We modeled the same floods over three different landscapes: the present-day topography and the GIA-corrected topography at 18 and 15.5 ka (Fig. 1*A* and *Methods*).

Rather than modeling flow from glacial lake Missoula to glacial Lake Columbia and then to the scabland tracts, we instead selected an inflow boundary through the center of glacial Lake Columbia, thus directly filling glacial Lake Columbia (green line; Fig. 1*A*). This simplification allowed us to focus our analysis on the effect of GIA on the scabland tracts downstream of glacial Lake Columbia, without additional complications from GIA affecting the routing upstream of glacial Lake Columbia. This simplification, however, might have impacted the model results. For instance, rather than a level initial water surface in glacial Lake Columbia that we modeled, the water surface might have been tilted by incoming water during high-magnitude flooding, which could have influenced flow partitioning between the tracts.

For all simulations, we set the initial glacial Lake Columbia water level to the highest observed lake level [730 m on present-day topography (13)], which corresponds to a time when Grand Coulee was blocked by ice or had not yet been carved. Although it is unlikely that the glacial Lake Columbia high stand persisted from 18 to 15.5 ka [estimated duration = hundreds of years (13)], and the lake level was likely different at different times, we used this same initial lake level for both time periods to isolate the influence of GIA on flood routing with all else held constant.

Multiple Missoula outburst floods are thought to have had a range of discharges (5, 9, 34) and flow durations. Even the order of magnitude of peak water discharge in individual flood channels is actively debated (9, 24). We focused on relatively modest flood events, which were selected to optimize the fit to erosional constraints imposed by the geographic distribution of scabland and loess-covered regions. To this end, we compared the modeled maximum bed shear stresses over the course of flooding (39 h; *Methods*) at every cell to mapped scabland regions where basalt was eroded by the floods and non-scabland regions that are loess covered (Fig. 1) (35). For basalt erosion, we used a threshold stress range of 117 to 1,242 Pa to initiate plucking, although alternative erosion mechanisms would require different shear stresses and thus different minimum discharges (24, 36) (*Methods* and *SI Appendix*, section 5). Channel discharge must have produced bed shear stresses surpassing this minimum threshold of 117 Pa in order to initiate incision in the scabland tracts. The loess erosion threshold is thought to be much smaller than for basalt [1 to 3 Pa (37)], although, in highly cohesive cases, threshold shear stresses could reach as high as 250 Pa (38). We assumed that if bed stresses in non-scabland loess-covered areas (including stream-lined loess areas, where some loess was eroded) surpassed the threshold for basalt plucking, the loess would have been rapidly eroded away (*Methods*).

For most simulations, we used a simple, triangular flood hydrograph with a peak spillover (input) discharge of 6 × 10^6^ m^3^/s that was reached after 10 h (total duration: 21 h, flooding in the scabland tracts analyzed over 39 h; *Methods*) from the glacial Lake Columbia inlet (Fig. 1*A*, green line and *Methods* and *SI Appendix*, Fig. S1). The peak discharge we modeled is within the range inferred by Clarke et al. (39) who modeled discharge via the formation of tunnels through the ice dam, but the flood is of shorter duration (21 h compared to 300 h). Other predictions with higher-peak discharges yield durations of ∼70 (16) and ∼140 h (6). The total water volume that enters glacial Lake Columbia in our model (464 km^3^) is smaller than the largest possible glacial Lake Missoula volume release [2,184 to 2,972 km (9, 16, 35)]. We explored the sensitivity of the results to flood discharge (5, 6, 7, and 10 × 10^6^ m^3^/s) and duration (21 to 34 h) (Table S1).

## Results

Loading of the Cordilleran ice sheet caused subsidence (relative to present-day elevation or equivalently modern, global mean sea surface elevation) north of the Channeled Scabland, and uplift (relative to present-day elevation) to the south on the ice sheet’s peripheral bulge (Fig. 1). At 18 ka, the Channeled Scabland region was uplifted ∼30 m more than the GIA-corrected topography at 15.5 ka (Fig. 1*B*). Both predictions of GIA-induced topographic change show a spatial elevation gradient between Telford-Crab Creek and Cheney–Palouse tracts (from ∼40 to 90 m at 18 ka; Fig. 1*B*; from ∼0 to 80 m at 15.5 ka; Fig. 1*C*). At 15.5 ka, there was a steeper elevation gradient caused by subsidence (relative to present-day elevation) to the northwest, near the Telford-Crab Creek tract, and uplift (relative to present-day elevation) in the southeast, near the Cheney–Palouse tract (Fig. 1*C*). This steeper elevation gradient resulted from isostatic adjustment to the growing Okanogan ice lobe, which was at its maximum extent in our ice history from 18 to 15 ka (*Methods*).

Floodwaters that entered the two scabland tracts were controlled by spillover regions [or low spots along the drainage divide (23)] from glacial Lake Columbia. We assigned the initial lake level, prior to flooding, to be the inferred, highest observed shoreline of 730 m (9, 40). We corrected this shoreline elevation for GIA at the French John’s locality (48.04° N, −118.69° W) (40) at 18 and 15.5 ka, resulting in initial lake levels of 790 and 748 m, respectively (*SI Appendix*, Fig. S2). GIA-corrected topographies are characterized by higher elevations compared to present day, with increasing elevation to the east (Fig. 1 *B* and *C*), resulting in a less extensive shoreline toward the east (*SI Appendix*, Fig. S2). Thus, GIA influenced the initial lake geometry and volume, and, by tilting the topography, it influenced both how the lake filled and where it overspilled.

Much of the input flood discharge went into raising the water level and flooding eastern regions of glacial Lake Columbia, resulting in substantially smaller, combined peak discharge entering both the Cheney–Palouse and Telford-Crab Creek tracts (0.7 to 3.1 × 10^6^ m^3^/s; *SI Appendix*, Fig. S3), as compared to the peak input discharge of 6 × 10^6^ m^3^/s. Even though our modeling simulated that the lake was minimally overspilling before ramping up the flood input discharge, the lake was able to impound more water during flooding. During the initial timesteps prior to flood input discharge ramp-up (0 to 5 h; *SI Appendix*, Fig. S1), overspilling from the initial lake level across spillover regions was an order of magnitude smaller than during peak flooding, reaching a maximum discharge of 1 and 2.5 × 10^5^, respectively, for the GIA-corrected and present-day topographies (*SI Appendix*, Fig. S3). Thus, lake levels initially fell slightly as the initial stage (730 m on present-day topography) drained prior to the input flood discharge ramp up. Flooding from increasing input discharge raised the water surface elevation in the lake, which greatly expanded the lake area and volume during the flood (*SI Appendix*, Fig. S2).

**Fig. 2.**
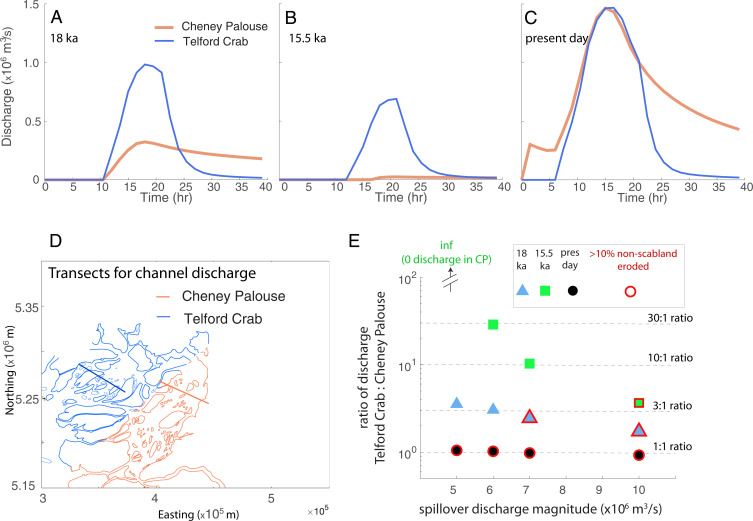
Channel discharge hydrograph for 18 ka (*A*), 15.5 ka (*B*), and present-day topography (*C*) for overspill peak discharge of 6 × 10^6^ m^3^/s (21 h duration) across transect for Cheney–Palouse (orange) and Telford-Crab Creek (blue), UTM zone 11 (*D*). (*E*) Ratio of peak channel discharge between Cheney–Palouse and Telford-Crab Creek (1 = equal discharge) for different peak spillover discharge magnitudes on GIA-corrected topography (blue triangles = 18 ka and green squares = 15.5 ka) and present-day topography (black circles). Red outlines indicate simulations in which more than 10% of non-scabland regions exceed the basalt erosion threshold. CP, Cheney-Palouse.

At 18 ka, parts of both tracts were flooded; however, about threefold greater discharge was routed into Telford-Crab Creek compared to Cheney–Palouse (Fig. 2 *A* and *E*) because uplift of the peripheral bulge generated higher elevations to the east (Fig. 1*B*). At 15.5 ka, the spatial gradient in uplift was amplified by subsidence due to the growth of the nearby Okanogan ice lobe, which was at its maximum extent in our ice history from 18 to 15 ka, lowering spillover elevations in Telford-Crab Creek relative to Cheney–Palouse. Thus, at 15.5 ka, about 30-fold greater discharge was routed into Telford-Crab Creek compared to Cheney–Palouse (Fig. 2 *B* and *E*). In contrast, simulations on non–GIA-corrected (present day) topography predicted that both tracts flooded simultaneously, with comparable discharges (Fig. 2 *C* and *E*). These results show how GIA substantially impacted the magnitude and partitioning of the simulated floods between the two tracts, both through tilting the tracts themselves and through its impact on the spillover elevations and the geometry of glacial Lake Columbia.

**Fig. 3.**
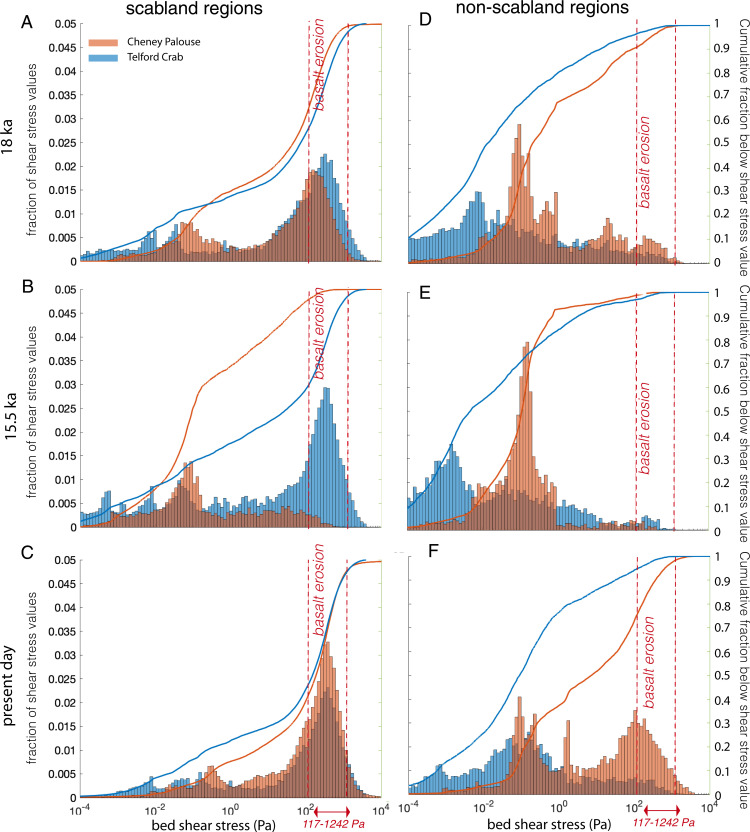
Histograms and empirical, cumulative distribution functions for maximum bed shear stresses in Telford-Crab Creek (blue) and Cheney–Palouse (orange) tracts for scabland regions (*Left; A–C*) and non-scabland regions (*Right; D–F*) for 6 × 10^6^ m^3^/s flood event on the 18 ka (*A* and *D*), 15.5 ka (*B* and *E*), and present-day (*C* and *F*) topography. Vertical dashed red lines show threshold shear stress values (117 to 1,242 Pa) required to erode basalt (see *Methods*).

For the 6 × 10^6^ m^3^/s (21 h) flood event on the 18-ka topography, both tracts were predicted to have eroded basalt with 35 and 44% of the terrain, with observed basalt erosion exceeding the threshold for basalt erosion in the Cheney–Palouse and Telford-Crab Creek tracts, respectively (Fig. 3*A* and *SI Appendix*, Fig. S4). In contrast, on the 15.5-ka topography due to loading of the Okanogan lobe, floodwaters were shifted toward the Telford-Crab Creek tract, and only 4% of scabland regions in Cheney–Palouse achieved stresses greater than the basalt erosion threshold, compared with 40% in the Telford-Crab Creek tract (Fig. 3*B* and *SI Appendix*, Fig. S4). For simulations on present-day topography, 52% of Telford-Crab Creek and 57% of Cheney–Palouse scabland regions exceeded the basalt-plucking threshold (Fig. 3*C* and *SI Appendix*, Fig. S4). Thus, the GIA-corrected simulations suggest that substantially more erosion in the Cheney–Palouse tracts occurred during earlier floods and that this sequencing of events was dictated by GIA.

Another constraint on flood discharges comes from considering the loess-covered terrain, which, through multiple floods, must have stayed loess covered. Consistent with this reasoning, results for the 6 × 10^6^ m^3^/s (21 h) flood event on the GIA-corrected topographies show that only a small fraction (9 and 1% for 18 and 15.5 ka, respectively) of the loess-covered (non-scabland) terrain would have experienced stresses in excess of the basalt erosion threshold. In contrast, for the same flood over the non–GIA-corrected (present day) topography, 24% of the loess-covered (non-scabland) terrain would have exceeded the threshold for basalt erosion (Fig. 3*F*). This comparison, therefore, increases our confidence that simulations on the GIA-corrected topographic reconstructions are more consistent with the observed spatial patterns of loess cover and basalt erosion than simulations on present-day topography (*SI Appendix*, Fig. S4).

To explore the influence of our flood hydrograph choice on our results, we simulated different hydrographs with a range in peak spillover discharges and flood durations. All tested hydrographs on GIA-corrected topographies resulted in a greater proportion of discharge in Telford-Crab Creek tracts relative to Cheney–Palouse tracts, particularly at 15.5 ka. The effect of GIA on discharge partitioning was less pronounced at higher discharges but was still substantial at the largest, simulated flood (Fig. 2*E*). For instance, for the highest, tested input discharge (10 × 10^6^ m^3^/s; duration = 21 h), there was 170% as much discharge in Telford-Crab Creek compared to Cheney–Palouse tracts at 18 ka and 350% as much discharge at 15.5 ka (Fig. 2*E* and *SI Appendix*, Fig. S5). The effect of GIA on flood partitioning was also reduced by increasing the flood duration to 34 h, resulting in ∼220% as much discharge in Telford-Crab Creek, compared to Cheney–Palouse tracts at 18 ka, and ∼560% as much discharge at 15.5 ka (*SI Appendix*, Fig. S5).

Simulations using the modern topography (Fig. 3*F*) and simulations with higher-peak discharges on GIA-corrected topography predicted that over 20% of the loess-covered (non-scabland) areas had bed stresses exceeding the basalt erosion threshold (Table S1), violating the constraint imposed by the geographic distribution of scabland regions. Thus, for the range in discharges (5 to 10 × 10^6^ m^3^/s) and flow durations (21 to 34 h) we simulated, the GIA-corrected flood simulations with a peak input discharge less than 7 × 10^6^ m^3^/s are more consistent with erosional constraints because they minimize the impact of flooding on the loess-covered terrain, as compared to the non–GIA-corrected simulations. A peak input discharge of 6 × 10^6^ m^3^/s (21 h) optimized fit to our erosional constraint, as it maximizes the extent of scabland basalt erosion (shear stress values >117 Pa) and minimizes basalt erosion (shear stress values <117 Pa) in non-scabland regions.

## Discussion

**Fig. 4.**
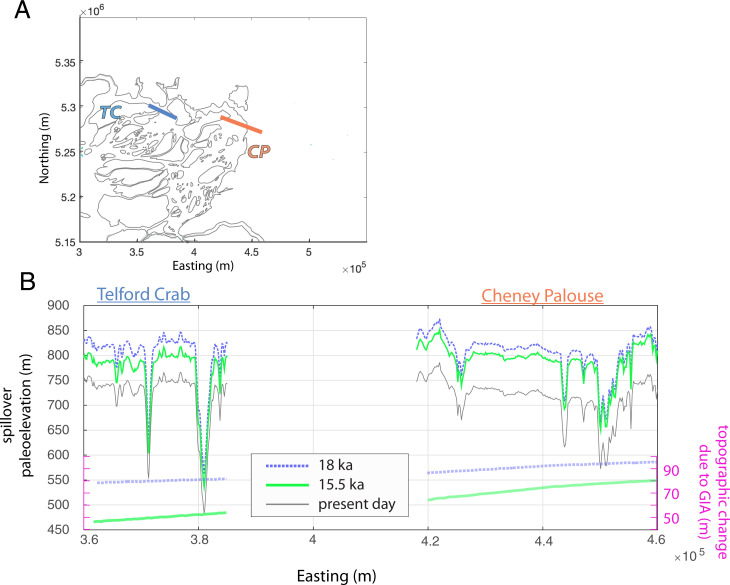
(*A*) Transect for Cheney–Palouse (CP; orange) and Telford-Crab Creek (TC; blue). (*B*) Paleoelevation profiles (*Left y*-axis) and topographic change due to GIA relative to present day (*Right y*-axis) for 18 ka (dashed blue), 15.5 ka (green), and present day (gray) across spillover transects in *A*: TC (blue) and CP (orange), UTM zone 11.

GIA directed how glacial Lake Columbia filled and spilled into scabland tracts from 18 to 15.5 ka by influencing the geometry of the lake, lake levels (Fig. 2*A* and *SI Appendix*, Fig. S2), and the paleoelevation of spillover regions (Fig. 4*B*). Although GIA impacts slopes across a broad spatial scale in the scabland tracts (Fig. 4*B*), its impact on spillover elevation is a more important control on discharge partitioning between the two tracts. Across all simulated floods, more discharge was partitioned into Telford-Crab Creek with GIA-corrected topographies, particularly at 15.5 ka, as compared to using the modern topography (Fig. 2*E* and *SI Appendix*, section 6).

The effect of GIA on flood partitioning partially depends on the discharge and duration of the flood events because these parameters influence the filling and overspill dynamics of glacial Lake Columbia. The ratio of discharge entering Telford-Crab Creek compared to Cheney–Palouse tracts became less pronounced with increasing flood discharge (Fig. 2*E*) owing to how the wetted cross-sectional areas in the spillover regions changed as a function of lake level (Fig. 4*B*). In addition, increasing input discharge directed more water into the Telford-Crab Creek and Cheney–Palouse tracts, rather than directing water into eastern regions of glacial Lake Columbia (*SI Appendix*, Fig. S6). At higher input discharges than those tested here, we might expect the influence of GIA to diminish as flood dynamics become less sensitive to the glacial Lake Columbia spillover elevations and channel geometries (Fig. 2*E*). Routing flood flow to Lake Columbia, rather than filling Lake Columbia directly, might also produce lake-filling and -overspilling dynamics that were not represented in our simplified, numerical experiments.

The peak spillover discharge we used (6 × 10^6^ m^3^/s; 21 h) resulted in shear stresses for a substantial fraction of the eroded basalt terrain (35 and 44% for the Cheney–Palouse and Telford-Crab Creek tracts, respectively, at 18 ka) that exceeded the threshold for basalt plucking, despite a smaller input flood volume compared to previous estimates of the largest possible Missoula flood events (6, 16). On average, the terrain might be expected to have evolved to a critical state where bed stresses were near the plucking threshold (24), which is qualitatively consistent with the distribution of stresses for the 6 × 10^6^ m^3^/s (21 h) flood event at 18 ka in both tracts and at 15.5 ka for the Telford-Crab tract (Fig. 3). While we expected that some regions had bed stresses that exceeded the plucking threshold during flooding, we would not expect this to be true everywhere. For instance, bedrock incision likely occurred by localized zones of erosion at knickzones, in flow convergences (41), or where rock was particularly fractured. These erosional zones would have moved through the scabland tracts like waves, leaving relatively stable topography in their wake (24).

Our simulations cannot directly explain the development of much of the scabland terrain within higher-elevation tracts that were not inundated by our simulated floods. One possible explanation is that those areas were eroded during larger floods that inundated the entire area (6) and that loess was far more resistant to erosion than we assumed, allowing for the inundated, loess-covered terrain to remain loess covered. A more likely possibility is that these higher-elevation areas were eroded before the topography evolved to its present state and then were isolated and abandoned during progressive incision and knickpoint migration as the topography evolved (24). Earlier floods, prior to the Last Glacial Maximum (9), would have incised scabland tracts under different flooding and GIA conditions. As the complicated network of scabland channels evolved, drainage overflow could have partitioned water between different flow pathways, allowing some areas to incise and capture flow, resulting in the abandonment of neighboring pathways (42–44).

## Conclusions

GIA affected the partitioning of Missoula floodwaters and, in doing so, dictated the simulated timing and extent of erosion within two major tracts in the Channeled Scabland. Future work should evaluate the impact of GIA in simulations that explore a wider range of flood hydrographs before conclusions can be reached about the exact erosional history of the Channeled Scabland. Nevertheless, our results highlight the potential for GIA to offer insight into erosional histories, providing a testable hypothesis for the spatiotemporal history of flood routing and erosion using emerging, geochronological constraints (18, 45). In turn, since landscapes are sensitive to Ice Age crustal deformation, this study underlines the utility of erosional histories in improving our understanding of past ice sheet size and geographic extent.

## Methods

### Glacial Isostatic Adjustment and Ice History Construction.

Calculating the response of the solid Earth to changes in ice loading requires an input for Earth’s viscoelastic structure and a history of global ice cover. We adopt an Earth model characterized by an elastic, lithospheric thickness of 48 km and an upper (48 to 670 km depth) and lower-mantle viscosity (670 to 2,891 km depth) of 0.3 × 10^21^ Pa/s and 7 × 10^21^ Pa/s, respectively, consistent with the lower range of mantle viscosity inferred from GIA analyses of North American deglaciation (46) and consistent with tomographic models, suggesting a thin, effective, lithospheric thickness and a lower-viscosity upper mantle in this region (47). We also considered the sensitivity of flooding simulations to GIA simulations using an alternate Earth model, VM2 (*SI Appendix*, Fig. S7), a frequently used Earth model largely based on Earth structure near Hudson Bay, Canada (48). Future work using 3D GIA modeling could improve the accuracy of our predictions by accounting for laterally variable mantle viscosity and lithospheric thickness across western North America (47, 49).

GIA-induced topographic changes in the Channeled Scabland are sensitive to changes in regional ice loading. Therefore, we performed GIA simulations using an ice loading history that captures both the growth and retreat of the Cordilleran ice sheet over the time period of Missoula megaflooding (22 to 14 ka). We constructed an ice loading history that incorporates geologic constraints on ice lobes near or in the Channeled Scabland region. We required that the Puget ice lobe reach its maximum extent in our ice history at 18 ka and retreats by 15 ka (50–52), although a recent study suggests that maximum extent may have occurred between 16 and 14.5 ka (53). For the Okanogan and Purcell lobes, ice advance began at 19 ka, and the lobes reached their maximum extent from 18 to 15 ka in our ice load reconstruction (18, 54). Although water depths in glacial Lake Missoula and glacial Lake Columbia were likely substantial, the lake diameters are relatively small (∼20 km) compared with the effective, lithospheric thickness (∼30 to 50 km) in this region (47). Furthermore, these lakes were potentially short lived (hundreds of years) compared to the many (1 to 10) kiloyears GIA response timescale (11, 13). Therefore, we did not include water loads in our GIA modeling.

While there are multiple, proposed North American ice sheet histories (46, 55, 56), we use the Australian National University (ANU) North American ice history as our base (46), as it resolves a detailed Cordilleran ice lobe geometry. We modified the ANU ice history to reflect these geologic constraints in the timing of local ice lobe advance and retreat. We require the maximum Okanogan ice lobe extent to occur from 18 to 15 ka. In addition, we require that the majority of melt of the North American ice sheet saddle, connecting the Cordilleran and Laurentide ice sheets, occurs from 13 to 11.5 ka, consistent with a recent, sea-level fingerprinting analysis of the Bering Strait flooding (28, 29). Because GIA-induced topographic change is sensitive to the prior ice loading history, our calculations span the time period from 58 ka to present. The ice loading history during the ice sheet growth phase (58 to 30 ka) is adopted from the ICE-PC2 history, characterized by a rapid growth of the eastern Laurentide ice sheet starting at 44 ka (57, 58). Since the ANU ice history is regional for North America, we include global ice sheets by adopting the ice history for the Scandinavian and Antarctic ice sheets from the ICE-6G ice history (56). We call this ice history GI31-ANUed-PC2 (*SI Appendix*, Fig. S8). We performed GIA calculations based on the theory and pseudo-spectral algorithm described by Kendall et al. (59), with a spherical, harmonic truncation at degree and order 256 (78-km resolution). Because of this coarse resolution, the associated ice history cannot be used to assess smaller-scale features of the glacial history, such as the timing of Columbia River blockage by ice.

Our GIA prediction, specifically the gradient in topographic change across the Channeled Scabland, is primarily sensitive to the local history of ice loading. However, the magnitude of uplift and subsidence is determined by the larger-scale loading history of the Cordilleran ice sheet, which has not been incorporated in earlier GIA corrections for the Channeled Scabland (9).

### Flood-Routing Simulations.

#### Initial conditions.

For ANUGA simulations on present-day topography, we used a 90-m resolution (vertical resolution = 16 m) digital elevation model from Shuttle Radar Topography Mission (Fig. 1*A*). This topography is characterized by a spatially uniform resolution with a maximum mesh triangle area of 250,000 m^2^ (0.25 km^2^ or ∼0.5-km resolution). We set the Manning’s roughness coefficient to 0.065, as in Larsen and Lamb (24), and explore the sensitivity to this choice in *SI Appendix*, Fig. S9. We adjusted present-day topography for GIA-induced topographic change by correcting for GIA at 18 and 15.5 ka. We also explored the age uncertainty on Columbia River damming by the Okanogan Lobe by performing simulations on topography corrected for GIA at 20 and 14 ka (*SI Appendix*, Figs. S10–S12).

We assigned an initial stage to the glacial Lake Columbia region to simulate spillover from a filled lake onto the Columbia Plateau. Glacial Lake Columbia is a topographically complex, drowned valley network, rather than a simple depression bounded by a plain. Thus, as the water level in the lake rises, the perimeter of the lake expands, and overspill locations can shift. We adopted the inferred highest lake level, 730 m (9, 40), on present-day topography simulations. We corrected this shoreline elevation for GIA at the French John’s locality (48.04° N, −118.69° W) (40) at 18 and 15.5 ka, resulting in initial stage levels of 790 and 748 m, respectively (*SI Appendix*, Fig. S2). We selected the highest recorded lake level of 730 in modeling spillover into the Channeled Scabland in our simulations, although prior studies suggest the stage of glacial Lake Columbia was lower than 730 m during most of its existence and may have only been at maximum stage for a few hundred years (13, 40). This lake stage corresponds to a time when Grand Coulee was blocked by ice or had not yet been carved.

#### Boundary conditions.

Floods were simulated in the region defined by the black, dashed white, and green lines in Fig. 1*A*. To approximate spillover discharge into glacial Lake Columbia, the inflow boundary conditions are defined by green lines in Fig. 1*A*, and discharge is required to flow from north to south across this boundary. The downstream boundary condition is set to a fixed water surface elevation, allowing water to exit at the three most southern ends of our domain (white dashed lines; Fig. 1*A*). The downstream water surface elevation is set to the elevation of topography. The downstream boundary location is far enough from our study area that our results are insensitive to the downstream boundary choice (*SI Appendix*, Fig. S13).

We simulated spillover discharge at the inlet (inflow) boundary using a simple flood hydrograph. The assigned discharge is distributed uniformly across inlet boundaries and represents spillover into the Channeled Scabland when glacial Lake Missoula outburst flooding entered a full glacial Lake Columbia. Although GIA likely influenced the distribution of water flowing into glacial Lake Columbia, and subsequently across its shorelines, we make the simplifying assumption that spillover discharge is uniform across the inlet boundary, which allows us to focus on how GIA impacts water routing once there is overspill from glacial Lake Columbia.

For each simulation, the input discharge is ramped up from zero in increments of 1 × 10^6^ m^3^/s from 1 × 10^6^ m^3^/s to the maximum peak discharge and then is ramped down with the same increments to zero over 21 h (*SI Appendix*, Fig. S1*A*). A peak discharge of 6 × 10^6^ m^3^/s is used in the main text and represents the highest modeled rate of spillover from glacial Lake Columbia (see Fig. 2 *A*–*D* for discharge in channel tracts, which is substantially smaller than input spillover discharge). We tested the sensitivity of our results to the prescribed hydrograph by running additional simulations using a maximum discharge of 5, 7, and 10 × 10^6^ m^3^/s (*SI Appendix*, section 6). We also varied the hydrograph duration by performing simulations with a peak discharge of 6 × 10^6^ m^3^/s, characterized by a longer duration (34 h compared to 21 h) (*SI Appendix*, Fig. S1*B* and section 6). We also performed a sensitivity test of our results to the open (outflow) boundary location by shifting the southernmost open boundary location from 3.5 × 10^5^ m Easting to 3.1 × 10^5^ m Easting (Universal Transverse Mercator [UTM] zone 11; *SI Appendix*, Fig. S13).

#### Model runs.

We ran flood simulations using topographies associated with the GI31-ANUed-PC2 ice history at 18 ka, 15.5 ka, and at present day. For each simulation, flood depths and velocities were analyzed over 39 h at 1.5-h increments (*SI Appendix*, Figs. S1 and S14). The flooding duration adopted in the main text results in peak discharge in Cheney–Palouse and Telford-Crab Creek after ∼20 h, similar to prior simulations which were based on releasing an initial lake volume of glacial Lake Missoula, which predicted that peak discharge in the Channeled Scabland was reached after 23 h (6).

### Calculating Erosion Thresholds.

Plucking of well-jointed Columbia River flood basalts formed the canyons of the Channeled Scabland, and the size of these basalt blocks can be up to ∼3 m in diameter (17, 24, 60, 61). Erosion by plucking can be dominated by downstream sliding of blocks (62). The threshold shear stress required for plucking was calculated in Larsen and Lamb (24) for Moses Coulee based on a set of values for bed angle, bed friction, block roughness, hydraulic lift force, and sidewall stress. The threshold shear stress for plucking via block sliding ranged from 117 to 1,242 Pa for the 16th and 84th percentile (D_16_ and D_84_) clast size from deposits in the Channeled Scabland (0.13- to 0.83-m diameter boulders in the Ephrata Fan and Drumheller Channels [see Extended Data Fig. 5 in Larsen and Lamb (24)]. Channel discharge must have produced bed shear stresses surpassing this minimum threshold of 117 Pa in order to initiate incision in the Channeled Scabland tracts.

We calculated the bed shear stress ($\tau_{b}$) in the flooded regions based on the simulated flooded water depth, *h*, and water velocities, ***u***, in both the x and y directions:

$$\tau_{b}=\rho c_{f}u^{2},$$

where $c_{f}$ is friction coefficient based on the Manning’s roughness coefficient *n*:

$$c_{f}=\frac{gn^{2}}{h^{1/3}}.$$

We used *n* = 0.065, as in Larsen and Lamb (24), and assume the roughness coefficient is spatially uniform. While the calculated shear stress values are sensitive to the choice of Manning’s friction coefficient (*SI Appendix*, Fig. S9), they are not sensitive to the choice of Earth model used in GIA simulations (*SI Appendix*, Fig. S7) or the open boundary location (*SI Appendix*, Fig. S13).

To compare erosional potential in the Cheney–Palouse and Telford-Crab Creek tracts, we compared shear stress values between these two regions (dashed white line; Fig. 1 *B* and *C*). We also compared shear stress values in regions where basalt erosion occurred against values in regions where no basalt erosion occurred. We used a surface geology map from the Washington State Geologic Survey (35) to identify basalt regions (scabland) and assumed these regions had experienced basalt erosion during megaflooding. The basalt regions indicate incision into the otherwise uneroded, loess-covered Columbia Plateau. The nonbasalt regions (non-scabland) are primarily loess covered, including minimally eroded, stream-lined loess regions, where floodwater flowed but did not erode to basalt. These loess deposits formed over the last 2 My (63).

## Supplementary Material

Supplementary File

## Data Availability

Source data for this research, including hydrodynamic modeling data, GIA-corrected topographic reconstructions, and analysis scripts, are publicly available on Zenodo (https://zenodo.org/record/5275157#.YfgvhPXML5Y). Source data have been deposited in Zenodo (https://zenodo.org/record/5275157#.YfgvhPXML5Y). All other study data are included in the article and/or *SI Appendix*.
