# Supplementary material for: Glacial isostatic adjustment directed incision of the Channeled Scabland by Ice Age megafloods
Source: Proc Natl Acad Sci U S A. 2022 Feb 14;119(8):e2109502119. doi: 10.1073/pnas.2109502119 (PMC8872708; doi:10.1073/pnas.2109502119)
Supplement: Supplementary File [file pnas.2109502119.sapp.pdf]

SUPPLEMENTARY MATERIAL

Supplementary Material for *Glacial isostatic adjustment directed incision of the Channeled Scabland by ice-age megafloods*

Authors: Pico. T., David, S.R., Larsen, I.J, Mix, A., Lehnigk, K., Lamb, M.P.

Source data for this research including hydrodynamic modeling data, glacial isostatic adjustment-corrected topographic reconstructions, and analysis scripts are publicly available on Zenodo (citable using the DOI: 10.5281/zenodo.5275157).

This supplementary material includes:

Supplementary Text, Section 1-6

Supplementary Figures 1-17

Supplementary Table 1

1. Timing constraints on ice history and flooding

Scouring of the Channeled Scabland likely occurred while the Columbia River was dammed by an advancing Okanogan ice lobe (Hanson, 1970; Waitt, 1985, 2017; Baker *et al.*, 2016; Balbas *et al.*, 2017), forming glacial Lake Columbia (Atwater, 1987; Hanson and Clague, 2016). Flood deposits dated to 18.2 $\pm$ 1.5 ka suggest that the Okanogan ice lobe blocked the Columbia River after this time, retreating after 15.4 $\pm$ 1.4 ka, based on the age of erratics, in addition to flood-transported boulders dated to 15.3-14 ka (Balbas *et al.*, 2017). Thus, glacial Lake Columbia likely existed after 18.2 and before 15.6 (1 sigma age uncertainty spans 19.7-14 ka); the Channeled Scabland were thus most recently carved during this time. Over eighty-nine flood events are recognized in varve sequences in glacial Lake Columbia, providing a minimum number of floods, given that other floods occurred before and after the blocking of Columbia River (Atwater, 1987; O'Connor *et al.*, 2020). Freshwater pulses in the ocean, as measured by δ^{18}O, show the largest freshwater event occurring between 20-18 ka, with other peaks at 16-15 ka, and 13-11.5

ka (Lopes and Mix, 2009; Praetorius *et al.*, 2020). The marine record is not sensitive to the source of freshwater, and may include various flood sources including Lake Bonneville, which encompassed a substantially larger lake volume than Lake Missoula (O'Connor *et al.*, 2020). Near British Columbia, 44 flood events with 80-50 yr periodicity from 19.3 to 14.9 ka were counted with sediments characteristic of Missoula floods (Cosma and Hendy, 2008; Gombiner *et al.*, 2016). Since we are interested in the formation of the Channeled Scabland on the Columbia Plateau, we focus on the time period when glacial Lake Columbia existed, from 18 to 15.5 ka, (or 20 to 14 ka, including age uncertainty).

2. *Uncertainties associated with GIA-corrected glacial Lake Columbia shoreline reconstruction*

To reconstruct glacial Lake Columbia extent and depth, the observed geologic shoreline elevation must be corrected for topographic change due to GIA. However, the shoreline age does not have sufficient age precision to differentiate between early (~18 ka) and later (~15.5 ka) floods (Atwater, 1987). Atwater (1987) suggested that the highest lake stage, 730 m, which we adopted in our simulations, may have only lasted for 200 to 400 years, however this estimate may represent a minimum since the record is incomplete. We chose the highest known lake level at both times to isolate the influence of glacial isostatic adjustment on spillover into the scabland tracts in our simulations. Nevertheless, the lake level associated with each time (18 ka and 15.5 ka) is a source of uncertainty in our performed simulations. Knowing the precise age of the lake shoreline would allow the appropriate GIA correction to be applied

to the observed shoreline elevation, which would help determine lake levels at the time of flooding. Thus, the initial lake level is an uncertainty in our flooding simulations since the most appropriate GIA correction to apply is unknown. Regardless of the shoreline's actual age, simulating megaflood overspilling from glacial Lake Columbia using present-day topography without correcting for GIA overestimates initial lake depths (Supplementary Figure 2).

3. Sensitivity Tests: Simulations at 20 ka and 14 ka

Floods at the earliest time within the age uncertainty (20 ka; Balbas *et al.*, 2017) on glacial Lake Columbia, simulated on the 20 ka GIA-corrected topography, result in comparable shear stress values between the channel tracts, similar to on the 18 ka topography for overspill discharge of $6x10^6$ m^3/s (21 hr) (Supplementary Figure 10; Supplementary Table 1). At the latest time within age uncertainty (14 ka), higher shear stress values are concentrated in the Telford-Crab Creek tract, similar to on the 15.5 ka topography simulations (Supplementary Figure 10; Supplementary Table 1).

4. Sensitivity tests: Earth model

We assessed the sensitivity of the calculated shear stress values to the earth model adopted in the GIA simulations. We adopted an alternate Earth model VM2 (Peltier, 2004) and modeled flooding on the 18 ka topography for overspill discharge of $6x10^6$ m^3/s (21 hr). We found that the resulting maximum shear stress values were insensitive to this choice (Supplementary Figure 7; Supplementary Table 1).

5. Sensitivity tests: Friction coefficient and threshold shear stress

We assessed the sensitivity of the calculated shear stress values to the choice of Manning's friction coefficient by performing ANUGA simulations and calculating shear stress values with a friction coefficient of n = 0.03, as compared to n = 0.065 (Supplementary Figure 9). Changing the value of the friction coefficient (keeping all other simulation parameters as in Figure 3) lowers the magnitude of calculated shear stress values (Supplementary Table 1). If a lower friction coefficient is more accurate for our study area, then shear stresses would be lower and a higher input spillover discharge would be required to produce maximum shear stress values exceeding the threshold to erode basalt.

Another uncertainty is the estimated threshold to erode basalt, which is based on entraining columnar basalt (Larsen and Lamb, 2016). In our study we assumed the majority of the basalt in the study region is columnar basalt. However, parts of this region are the top of basalt flow, or entablature basalt (Baker, 1978; Baker *et al.*, 2016). For eroding entablature basalt, thresholds for plucking may be greater than assumed in our study, as prior literature suggests entablature basalt is more resistant to erosion that underlying columns (Schultz, 1995; Baker *et al.*, 2016). Another possibility is that lower shear stress thresholds are required for plucking than for transport. Long, skinny, loose columns might be plucked from a ledge more easily than they could be transported along the bed.

The channelized landscape is used in our comparison with the threshold shear stress for basalt erosion. However, where basalt columns were plucked above a threshold shear stress, rock that could be eroded at shear stresses below that threshold would now be absent, altering channel geometry such that later flood events would produce lower shear stresses (e.g. Lapotre and Lamb, 2015). If substantial erosion occurred during flooding, the post-erosion state would have a higher erosion threshold since exposed, loose columns would have been removed, leaving the less-weathered top of the underlying lava flow. Thus, simulations using the post-erosion state, which is what we include as the initial condition for topography in our simulations, may require higher shear stress thresholds than we assumed.

6. Distribution of flood discharge and sensitivity to input discharge

We analyzed the distribution of spillover discharge into four cross-sections bordering glacial Lake Columbia across the entire study area. Supplementary Figure 3 shows the discharge evolution for 18 ka (Supplementary Figure 3A), 15.5 ka (Supplementary Figure 3B), and present-day topography (Supplementary Figure 3C) for overspill discharge of $6x10^6$ m^3/s (21 hr) across all regions of the study area. Discharge entering both the Telford-Crab Creek and Cheney-Palouse tracts (blue; Supplementary Figure 3) varies depending on the topography used in simulations. On the 18 ka GIA-corrected topography, the maximum discharge entering Telford-Crab Creek and Cheney-Palouse is $1.4x10^6$ m^3/s, in contrast to $0.7x10^6$ m^3/s on the 15.5 ka GIA-corrected topography. Simulations using present-day topography result in a higher maximum discharge of $3x10^6$ m^3/s. These differences result both from differing initial lake stages for 18 ka

and 15.5 ka GIA-corrected topographies and present-day topography (Supplementary Figure 2), as well as differing spillover elevations on each topography (Figure 4C).

The spillover discharge entering the eastern region of the study area (red and yellow; Supplementary Figure 3) contributes to filling and expanding the extent of glacial Lake Columbia, as this region is inundated. The discharge evolution through these cross sections experiences an initial spike in the first timestep of the simulation on the order of 0.1-0.2x10^6 m^3/s. This initial discharge peak is from outflow of the initial lake stage, rather than the input spillover discharge.

To explore how the calculated maximum shear stresses in the main text vary with maximum input spillover discharge magnitude, we performed simulations with a lower and higher maximum discharge of 5 x10^6 m^3/s (21 hr) and 7 x10^6 m^3/s (21 hr), respectively. Supplementary Figure 15 and Supplementary Figure 16 show the associated histograms and empirical cumulative distribution functions for maximum bed shear stresses compared with the basalt erosional threshold. At 15.5 ka, a peak spillover discharge of 5x10^6 m^3/s (21 hr) results in exceeding the threshold to erode basalt in Telford-Crab Creek, but not in Cheney-Palouse (Supplementary Table 1). Because subsidence due to glacial isostatic adjustment concentrates erosional power in Telford-Crab Creek at 15.5 ka, a smaller discharge than that used in the main text can erode this single tract.

We assessed the sensitivity of our simulation results to different input spillover discharge scenarios. We performed simulations with a longer flood duration with a maximum discharge of 6 $x10^6$ m^3/s, with a longer duration (34 hrs) input discharge floodwave than that used in the main text. We also performed simulations using a higher maximum discharge of 10 $x10^6$ m^3/s (21 hr). Supplementary Figure 5 shows the discharge evolution in Telford-Crab Creek and Cheney-Palouse channel tracts for each of these discharge scenarios. We found similar patterns of discharge partitioning between Telford-Crab Creek (blue; Supplementary Figure 5) and Cheney-Palouse (orange; Supplementary Figure 5) to the results from simulations adopted in the main text. In particular, more discharge is routed into Telford-Crab Creek tracts relative to Cheney-Palouse on the 18 ka GIA-corrected topography compared (Supplementary Figure 5A) to on the 15 ka GIA-corrected topography (Supplementary Figure 5B). For simulations on the present-day topography, an equivalent discharge entered both channel tracts (Supplementary Figure 5C).

Although these sensitivity tests suggest our major result (GIA influences discharge distributions) is robust, discharge partitioning between Telford-Crab Creek and Cheney-Palouse may change under substantially different flooding scenarios. Telford-Crab Creek and Cheney-Palouse tracts are characterized by different channel geometries; in particular, channels are narrower in Telford-Crab Creek compared to in Cheney-Palouse (Figure 4C). Thus, with increasing magnitudes of discharge, we would expect a greater volume in the Cheney-Palouse tract, where channel cross-sectional areas are greater. At very high discharges, we may expect the influence of glacial isostatic adjustment to diminish as flood dynamics become less sensitive to the

geometry of individual channels in these tracts. Indeed, Supplementary Figure 5 shows that the proportional difference in discharge entering Telford-Crab Creek and Cheney-Palouse tracts becomes less pronounced at the higher spillover discharge rate of 10 x10^6 m^3/s (21 hr).

We explored how the ratio of discharge entering Telford-Crab Creek and Cheney-Palouse tracts varied at different input spillover discharge magnitudes (Figure 2E). Simulating spillover from glacial Lake Columbia on GIA-corrected topographies results in a greater proportion of discharge in Telford-Crab Creek tracts relative to Cheney Palouse tracts, particularly at 15.5 ka. The influence of GIA on discharge partitioning becomes less pronounced at higher discharges. However, at the higher tested spillover discharges, more than 10% of non-scabland regions are predicted to exceed the threshold to erode basalt, violating the constraint imposed by non-scabland areal extent (Supplementary Figure 17; Supplementary Table 1).

We also analyzed the distribution of spillover discharge into four cross-sections bordering glacial Lake Columbia across the entire study area (as in Supplementary Figure 3 for main text simulations) using these additional flood discharge scenarios. Supplementary Figure 6 shows the discharge evolution for 18 ka (Supplementary Figure 6A), 15.5 ka (Supplementary Figure 6B), and present-day topography (Supplementary Figure 6C). In contrast to simulations adopted in the main text, greater peak spillover discharge results in sufficient backfilling of glacial Lake Columbia to allow a greater proportion of spillover discharge to enter the Telford-Crab Creek and Cheney-Palouse tracts (blue; Supplementary Figure 6) after ~15 hours.

SUPPLEMENTARY FIGURES

Supplementary Table 1: Percentage of area with maximum shear stresses that exceed 117 Pa for each flood simulation. Bold text highlights simulations where more than 10% of non-scabland area exceeds basalt erosion threshold. TC is Telford-Crab Creek and CP is Cheney-Palouse.

		**scabland (basalt-scoured) regions**		**non-scabland (loess-covered) regions**	
**simulation**	**peak discharge ($x10^6$ m^3/s)**	TC	CP	TC	CP
present day	5	41.90%	49.14%	3.47%	**17.79%**
18 ka	5	31.70%	22.16%	1.15%	5.18%
15.5 ka	5	19.47%	0.00%	0.07%	0.00%
present day	*(main text)* 6	52.34%	56.85%	5.24%	**24.55%**
18 ka	*(main text)* 6	43.82%	35.46%	3.44%	9.13%
15.5 ka	*(main text)* 6	40.21%	4.29%	3.16%	1.32%
present day	7	60.48%	64.57%	8.88%	**31.05%**
18 ka	7	54.41%	45.72%	7.55%	**14.41%**
15.5 ka	7	49.61%	22.82%	7.19%	5.34%
present day	10	66.82%	74.35%	**18.26%**	**42.21%**
18 ka	10	67.97%	67.29%	**19.53%**	**33.16%**
15.5 ka	10	68.08%	52.27%	**22.65%**	**20.02%**
present day	6, 34 hrs	62.32%	66.64%	**10.20%**	**32.36%**
18 ka	6, 34 hrs	59.98%	51.36%	**11.24%**	**19.83%**
15.5 ka	6, 34 hrs	57.43%	35.78%	**12.56%**	9.72%
boundary condition test 18 ka	6	43.20%	35.83%	2.96%	8.97%
alternate earth model 18 ka	6	54.69%	47.22%	8.85%	**14.80%**
alternate friction coef 18 ka	6	14.40%	6.29%	0.60%	1.85%
20 ka	6	46.44%	38.59%	4.12%	**10.34%**
14 ka	6	40.99%	18.24%	3.13%	4.53%

A.

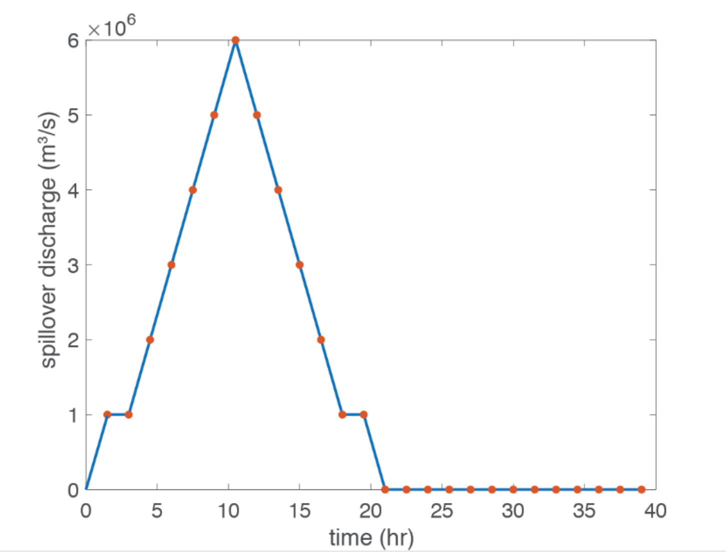
×10^6
spillover discharge (m^3/s)
0 1 2 3 4 5 6
0 5 10 15 20 25 30 35 40
time (hr)

B.

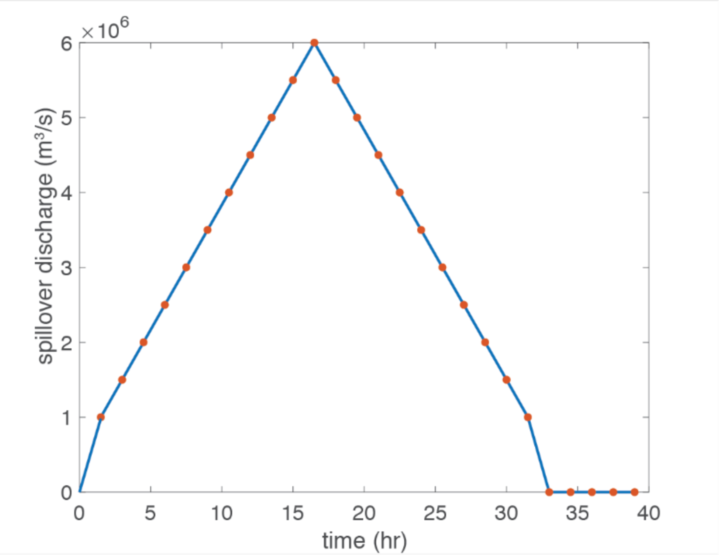
×10^6
spillover discharge (m^3/s)
0 1 2 3 4 5 6
0 5 10 15 20 25 30 35 40
time (hr)

C.

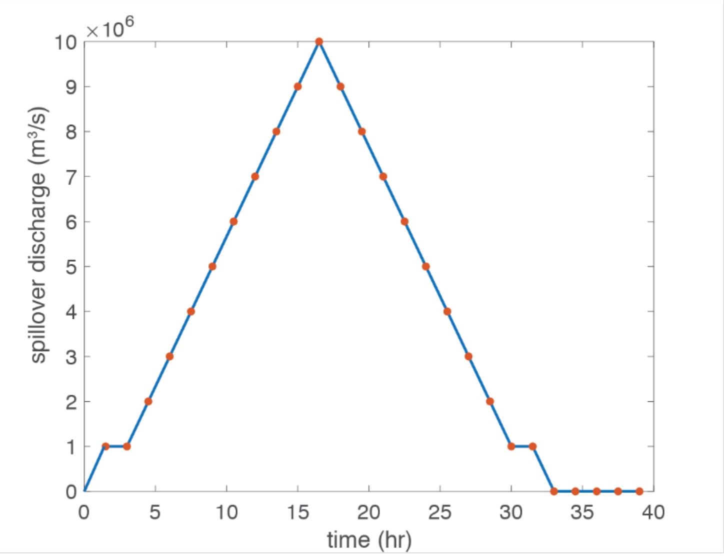
×10^6
spillover discharge (m^3/s)
0 1 2 3 4 5 6 7 8 9 10
0 5 10 15 20 25 30 35 40
time (hr)

Supplementary Figure 1| Inlet spillover discharge from glacial Lake Columbia hydrograph for simulations in main text with maximum discharge of $6x10^6$ m^3/s (21 hr) (A) and $6x10^6$ m^3/s with twice the duration as the floodwave duration in the main text (34 hr) (B), and $10x10^6$ m^3/s (21 hr) (C).

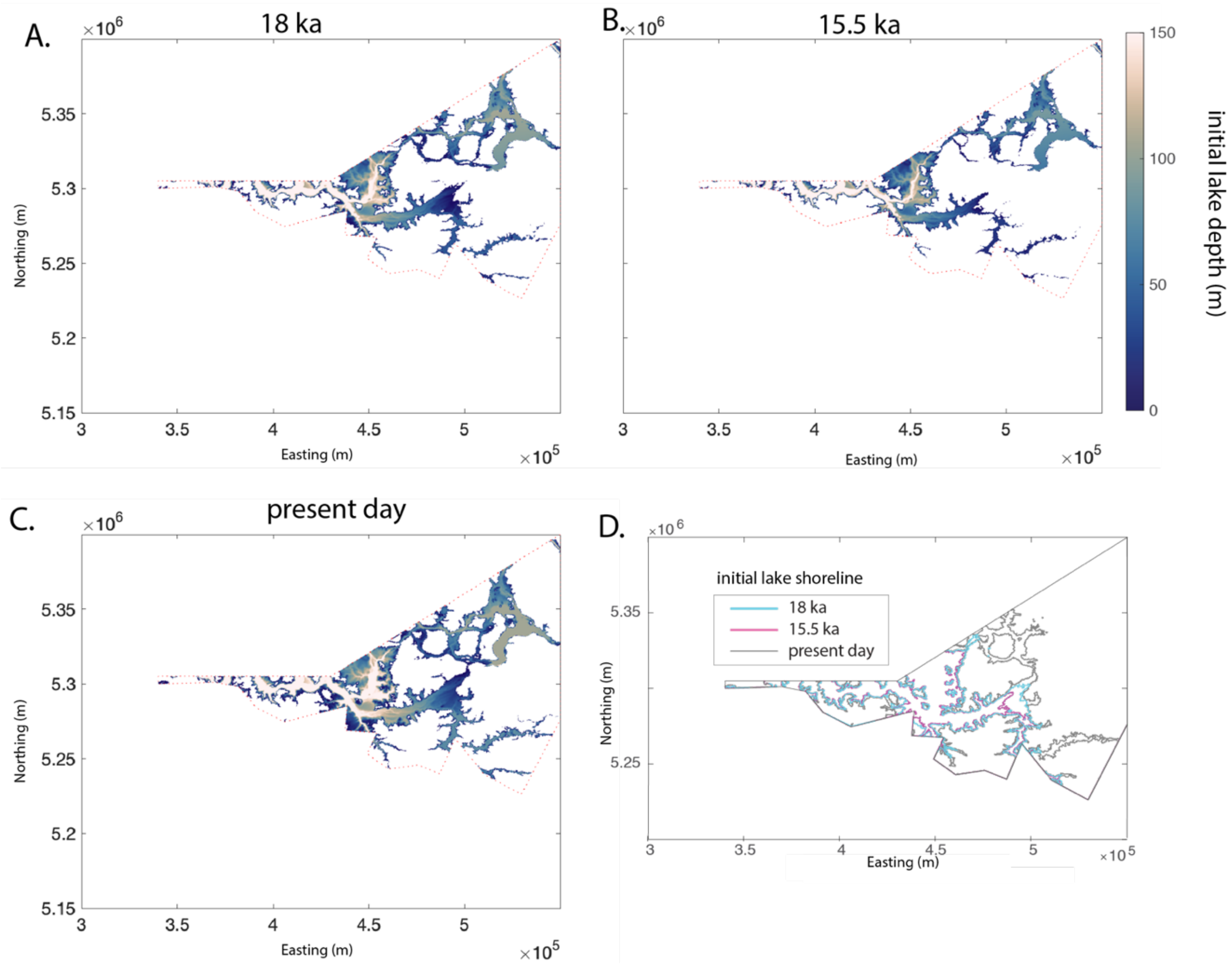

Supplementary Figure 2 | glacial Lake Columbia initial lake depth at (A) 18 ka, (B) 15.5 ka, and (C) present day. Dotted red lines show outline of the boundary where topography is filled to glacial Lake Columbia maximum lake stage (730 m for present day, 748 m for 15.5 ka, and 790 m for 18 ka). D. Comparison of initial lake shorelines for 18 ka (blue), 15.5 ka (pink), and present-day (black) topographies. The 18 ka and 15.5 ka glacial Lake Columbia boundaries do not connect in the northeastern corner of the modeled domain.

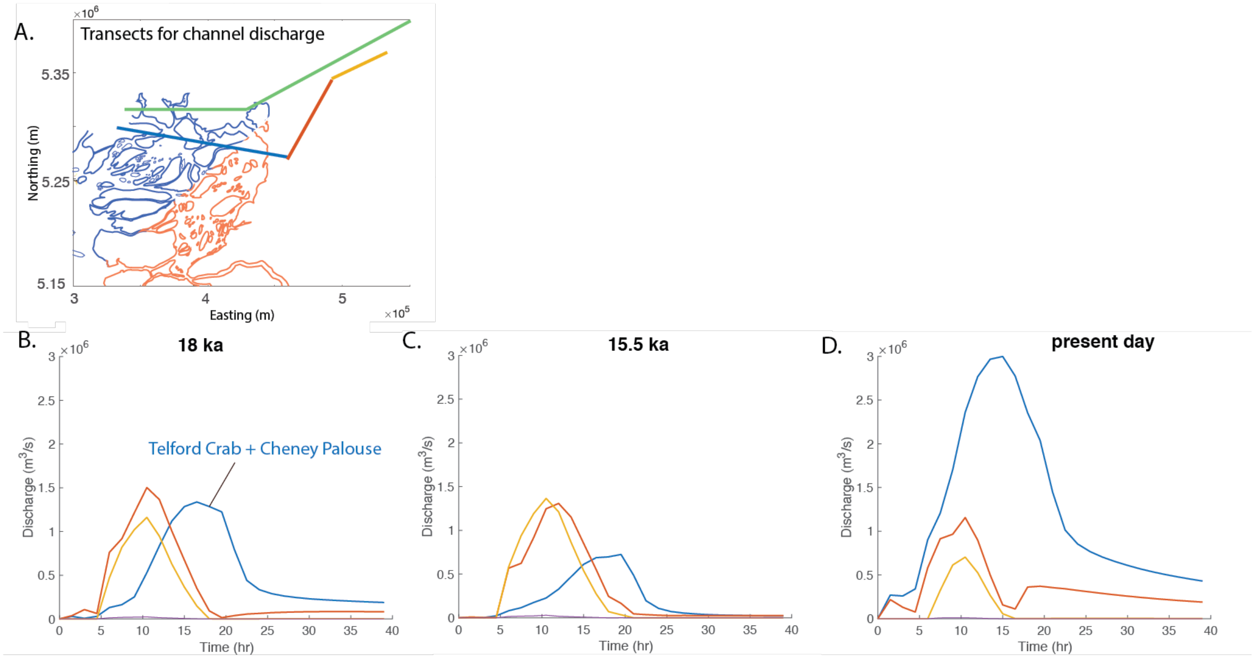

Supplementary Figure 3| Map of cross section locations (A), including inlet boundary in green (as in Figure 1A). Discharge evolution for 18 ka (B), 15.5 ka (C), and present-day topography (D) for overspill peak discharge of $6x10^6$ m^3/s (21 hr) across all regions of study area. The blue line represents a cross section of discharge entering the Channeled Scabland region including Telford-Crab Creek and Cheney-Palouse. Yellow and red lines represent cross sections entering the eastern region of the study area, which contribute to filling and expanding the extent of glacial Lake Columbia.

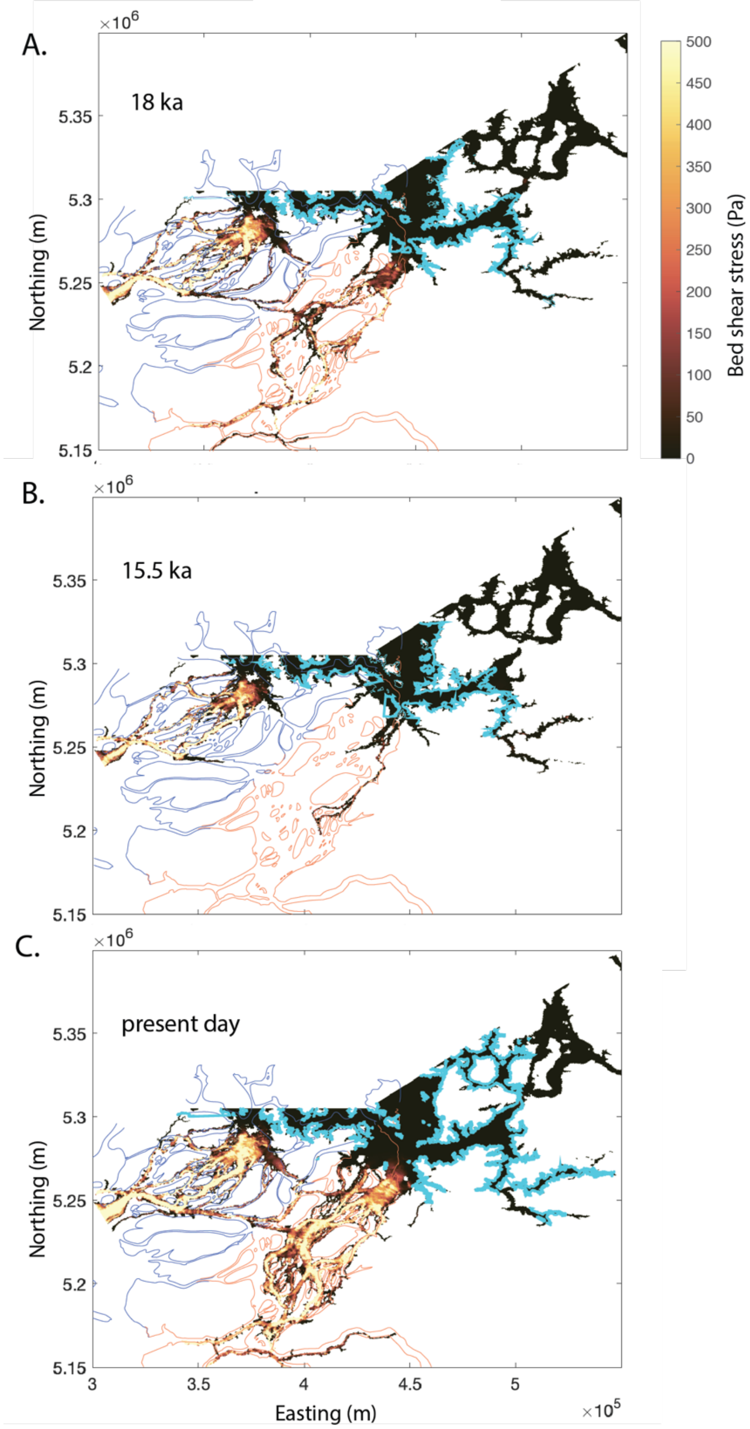

Supplementary Figure 4 | Maximum achieved bed shear stress over course of simulation (see Methods) on 18 ka GIA-corrected (A), 15.5 ka GIA-corrected (B), present-day (C) topography with a peak discharge of 6x10^6 m^3/s (21 hr). Orange (Cheney-Palouse) and blue (Telford-Crab Creek) lines contain basalt-eroded regions (*Washington State*

Geologic Survey Surface Geology Map 1:100,000, 2020). Turquoise lines indicate glacial Lake Columbia shoreline at start of simulation. UTM zone 11.

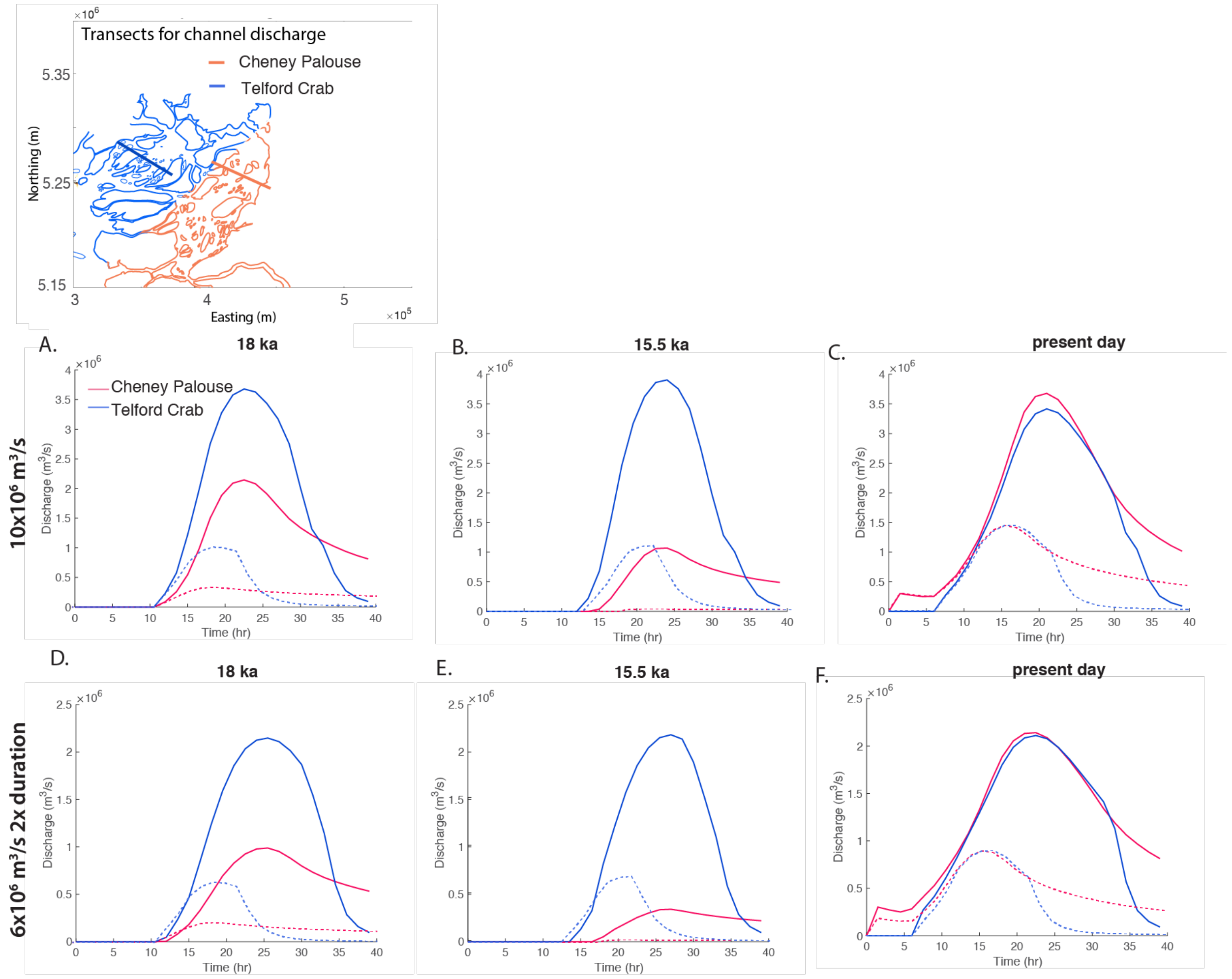

Supplementary Figure 5 | Solid lines show discharge evolution for 18 ka (A,D), 15.5 ka (B,E), and present-day topography (C,F) in Telford-Crab Creek and Cheney-Palouse tracts for overspill discharge of $10x10^6$ m^3/s (top), and $6x10^6$ m^3/s with twice the duration (34 hr) as the floodwave duration in the main text (bottom). Dashed lines show comparison to discharge evolution in Figure 2 for $6x10^6$ m^3/s (21 hr) flood event.

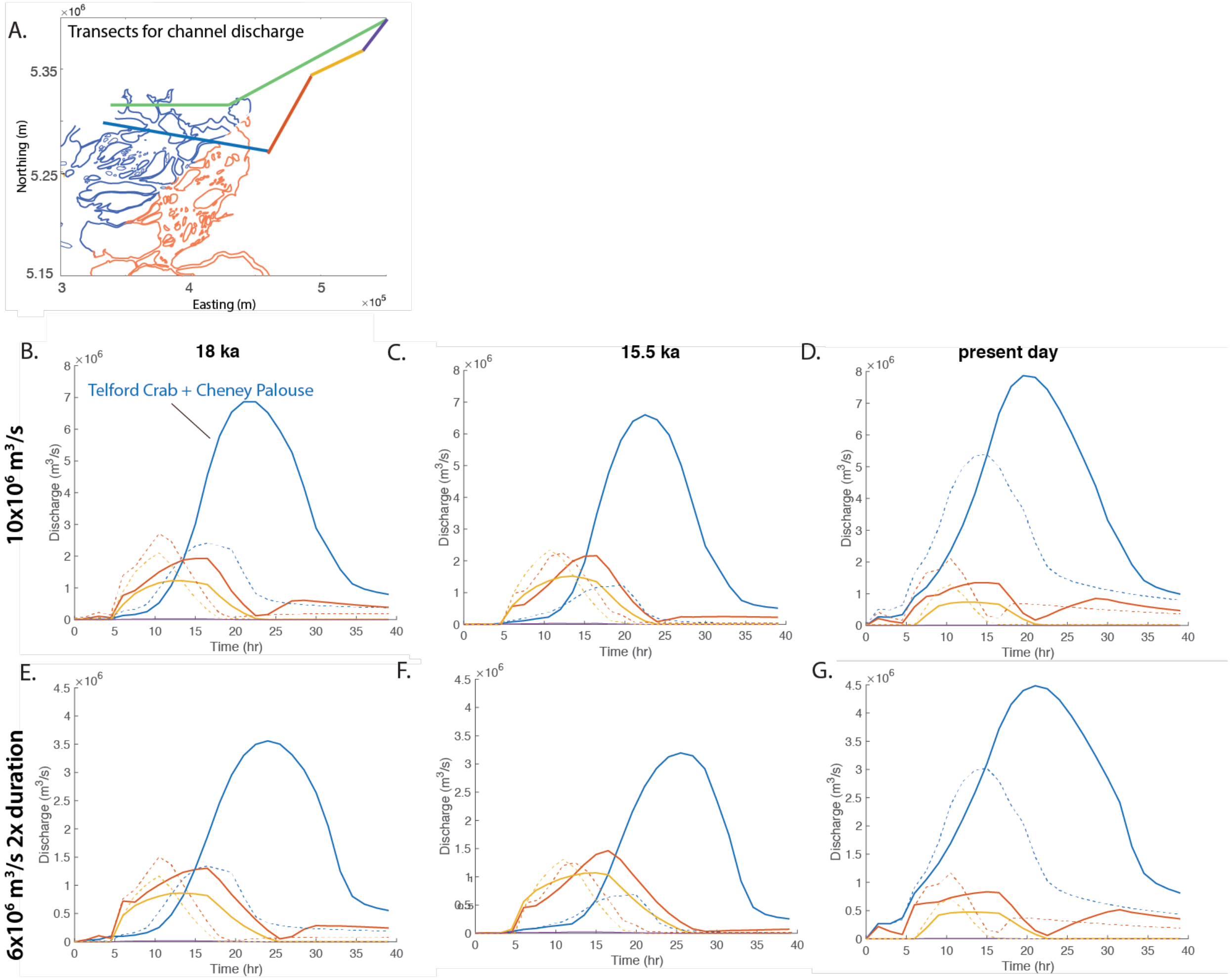

Supplementary Figure 6| Map of cross section locations (A), including inlet boundary in green (as in Figure 1A). Discharge evolution for 18 ka (B,E), 15.5 ka (C,F), and present-day topography (D,G) for overspill discharge of $10x10^6$ m^3/s (21 hr) (top), and $6x10^6$ m^3/s with twice the duration (34 hr) as the floodwave duration in the main text (bottom). The blue line represents a cross section of discharge entering the Channeled Scabland region including Telford-Crab Creek and Cheney-Palouse. Yellow, red, and purple lines represent cross sections entering the eastern region of the study area, which contribute to filling and expanding the extent of glacial Lake Columbia. Dashed lines show comparison to discharge evolution in Supplementary Figure 3 for $6x10^6$ m^3/s (21 hr) flood event.

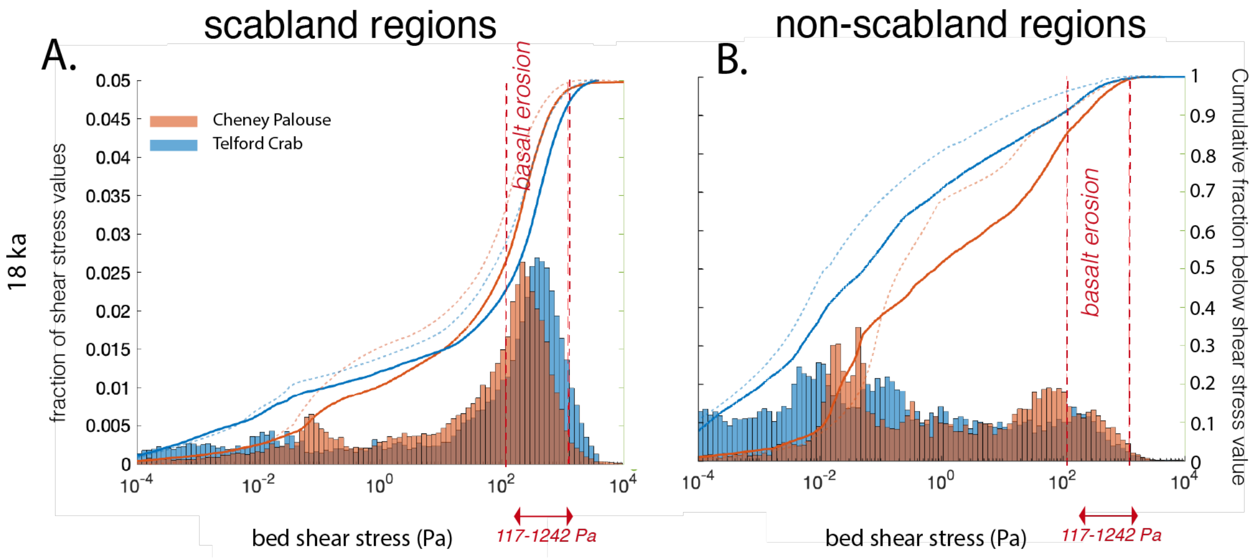

Supplementary Figure 7 | Histograms and empirical cumulative distribution functions for maximum bed shear stresses in Telford-Crab Creek (blue) and Cheney-Palouse (orange) for alternate Earth model VM2 on 18 ka topography (all other parameters held constant, using peak spillover discharge of 6x10^6 m^3/s (21 hr) as in Figure 4). for (A) scabland regions and (B) non-scabland regions. Vertical dashed red lines show threshold shear stress values (117-1242 Pa) required to erode basalt. The dashed blue and orange lines show the empirical cumulative distribution functions in main text Figure 3.

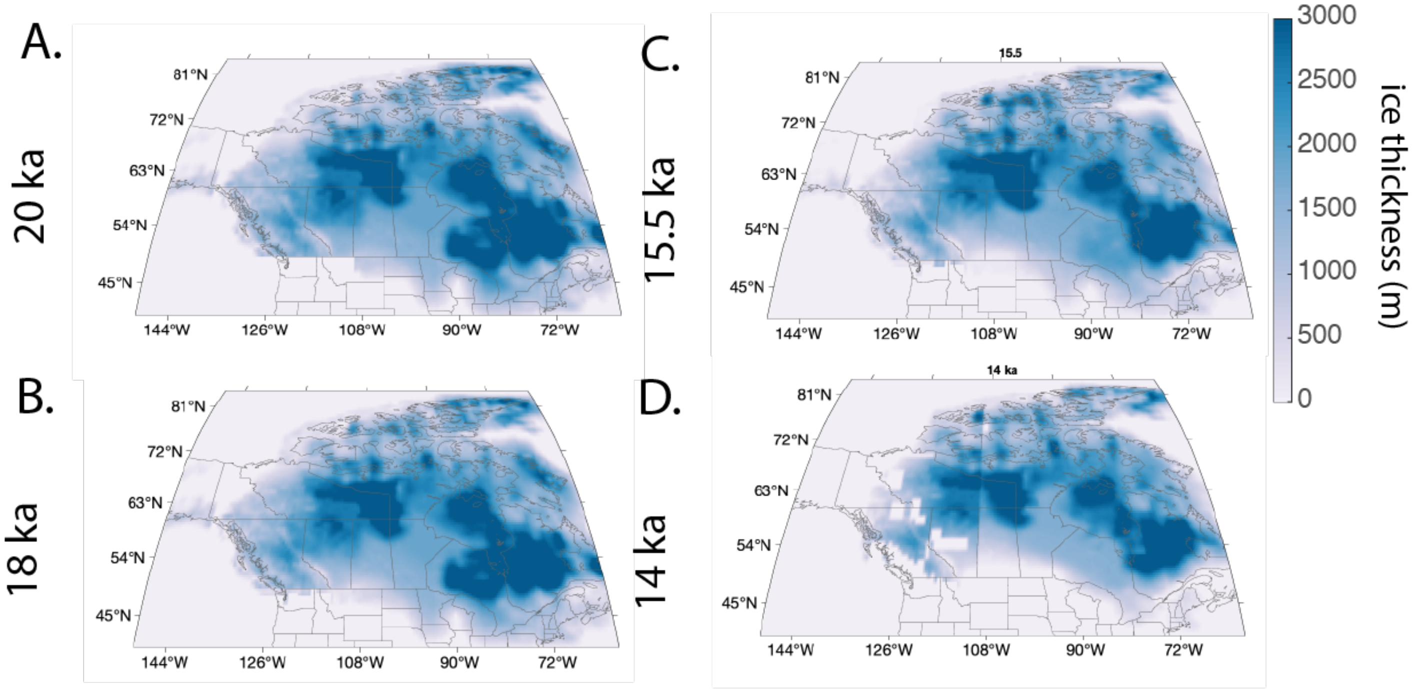

Supplementary Figure 8 | GI31-ANUed-PC2 North American ice thickness at 20 ka (A), 18 ka (B), 15.5 ka (C), and 14 ka (D).

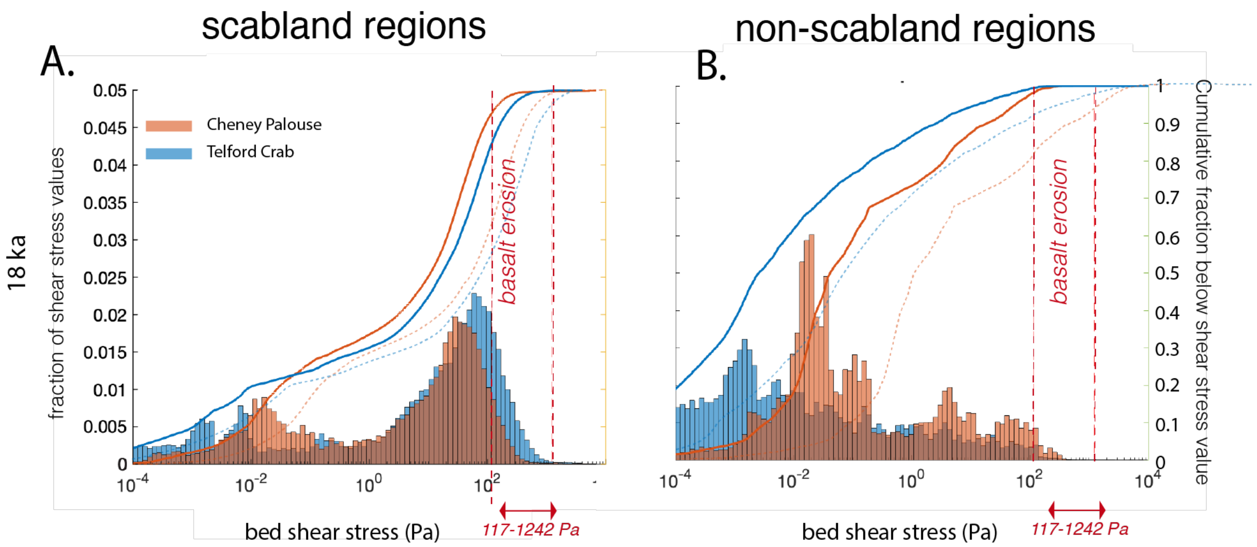

Supplementary Figure 9| Histograms and empirical cumulative distribution functions for maximum bed shear

stresses in Telford-Crab Creek (blue) and Cheney-Palouse (orange) for simulations using Manning's coefficient n = 0.03 with a discharge of 6x10^6 m^3/s (21 hr) on the 18 ka topography for (A) scabland regions, (B) non-scabland regions. Compare to simulations shown in Figure 4 (n = 0.065, all other parameters held constant). The dashed blue and orange lines show the empirical cumulative distribution functions in main text Figure 3.

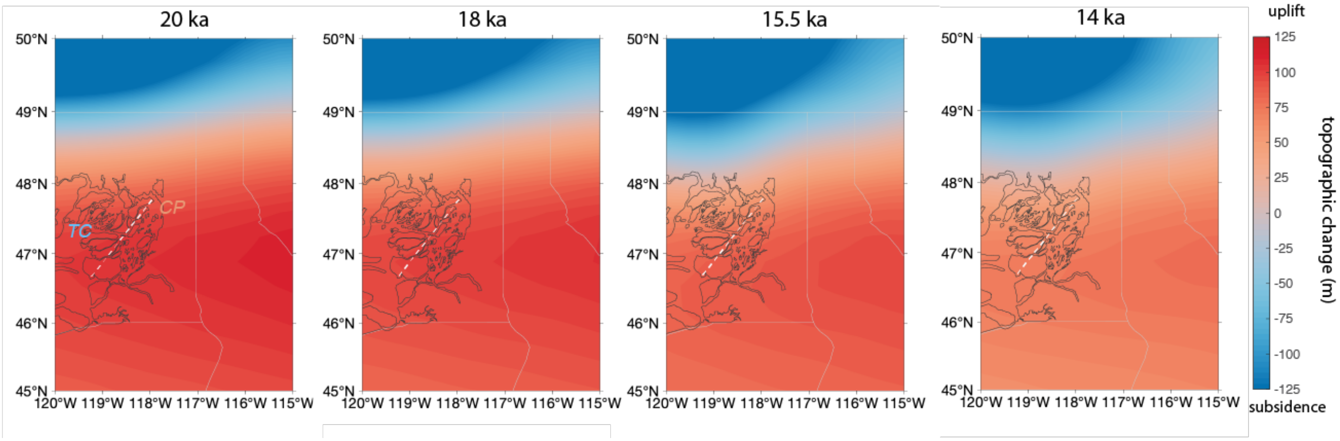

Supplementary Figure 10 | Topographic change due to glacial isostatic adjustment with ice history GI31-ANUed-PC2 for 20-14 ka

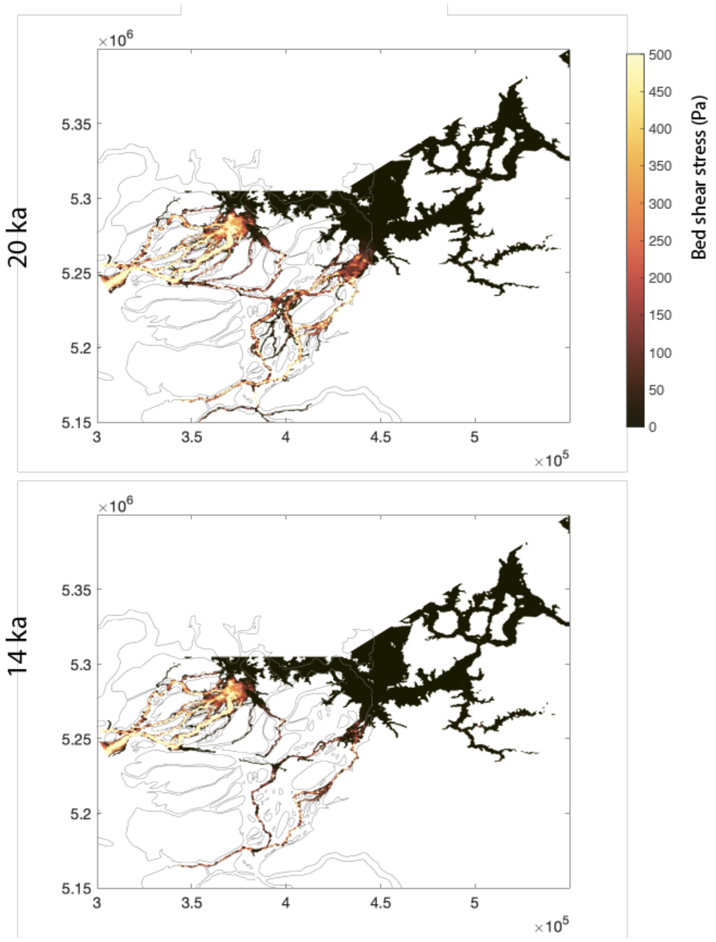

Supplementary Figure 11 | Maximum shear stress on 20 ka (A) and 14 ka (B) topography for simulations with same parameters as in Supplementary Figure 4, using discharge of 6x10^{6} m^{3}/s (21 hr). Gray lines contain basalt-eroded regions (*Washington State Geologic Survey Surface Geology Map 1:100,000*, 2020).

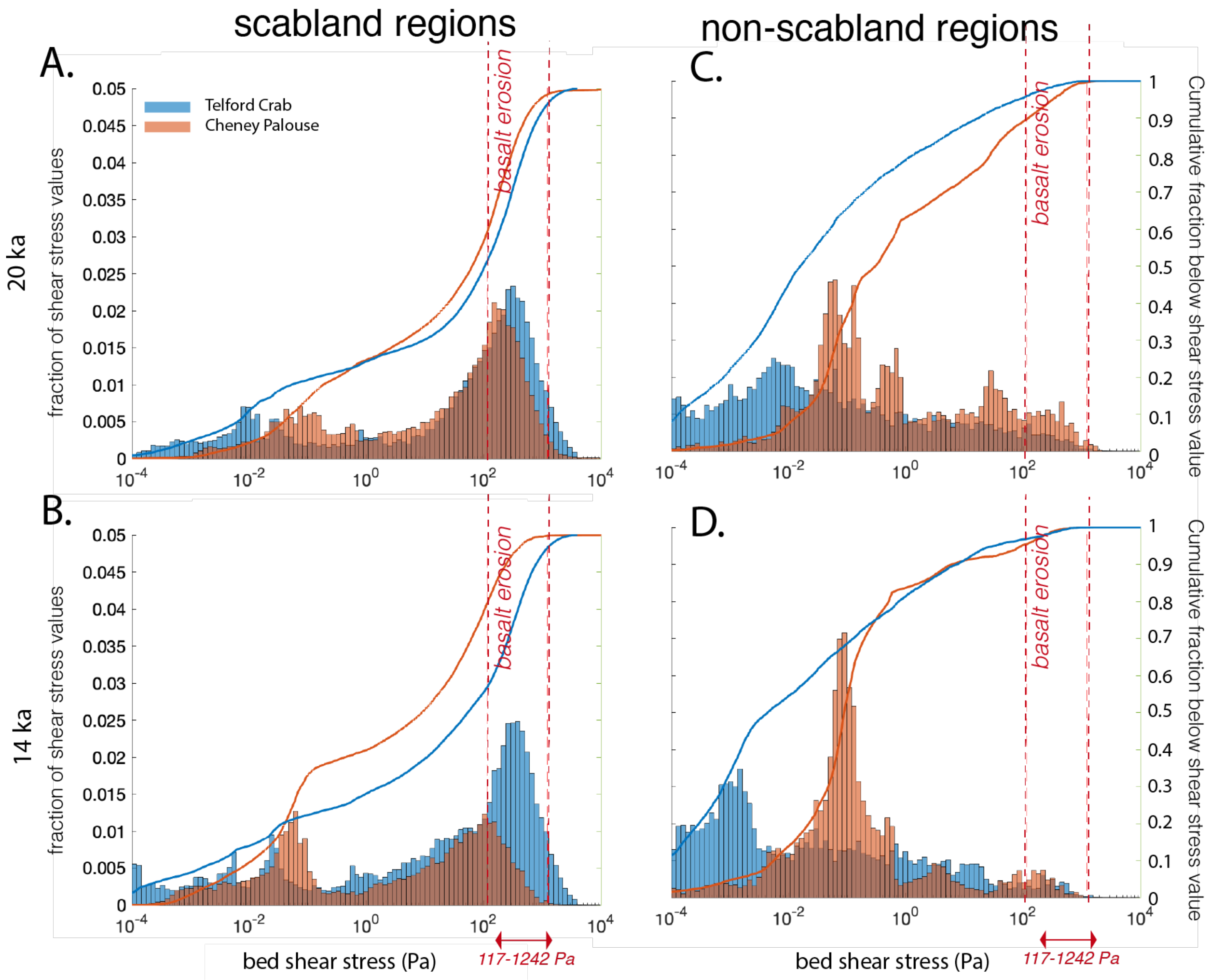

Supplementary Figure 12| Histograms and empirical cumulative distribution functions for maximum bed shear stresses in Telford-Crab Creek (blue) and Cheney-Palouse (orange) using peak spillover discharge of 6x10^6 m^3/s (21 hr), as in Figure 4, for 20 ka (A,C) and 14 ka topography (B,D) for scabland regions (A,B) and non-scabland regions (C,D). Vertical dashed red lines show threshold shear stress values (117-1242 Pa) required to erode basalt

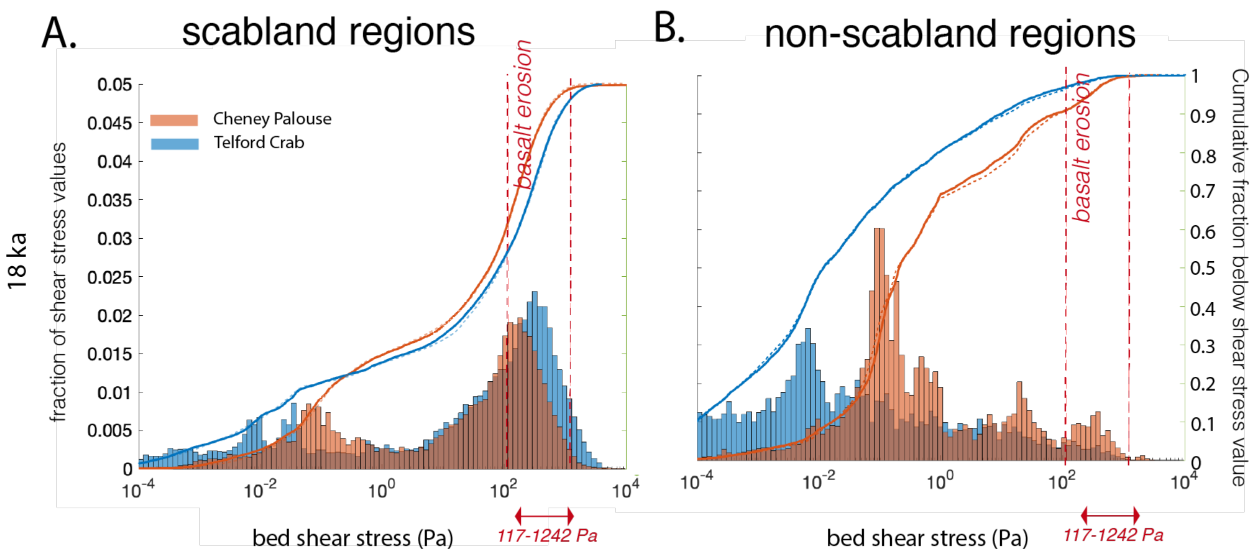

Supplementary Figure 13| Histograms and empirical cumulative distribution functions for maximum bed shear stresses in Telford-Crab Creek (blue) and Cheney-Palouse (orange), (A) in scabland regions and (B) non-scabland regions, for alternate downstream boundary condition. In this simulation the southernmost open boundary location is shifted from $3.5x10^5$ m Easting to $3.1x10^5$ m Easting (all other parameters held constant, using peak spillover discharge of $6x10^6$ m^3/s (21 hr) as in Figure 4). Vertical dashed red lines show threshold shear stress values (117-1242 Pa) required to erode basalt

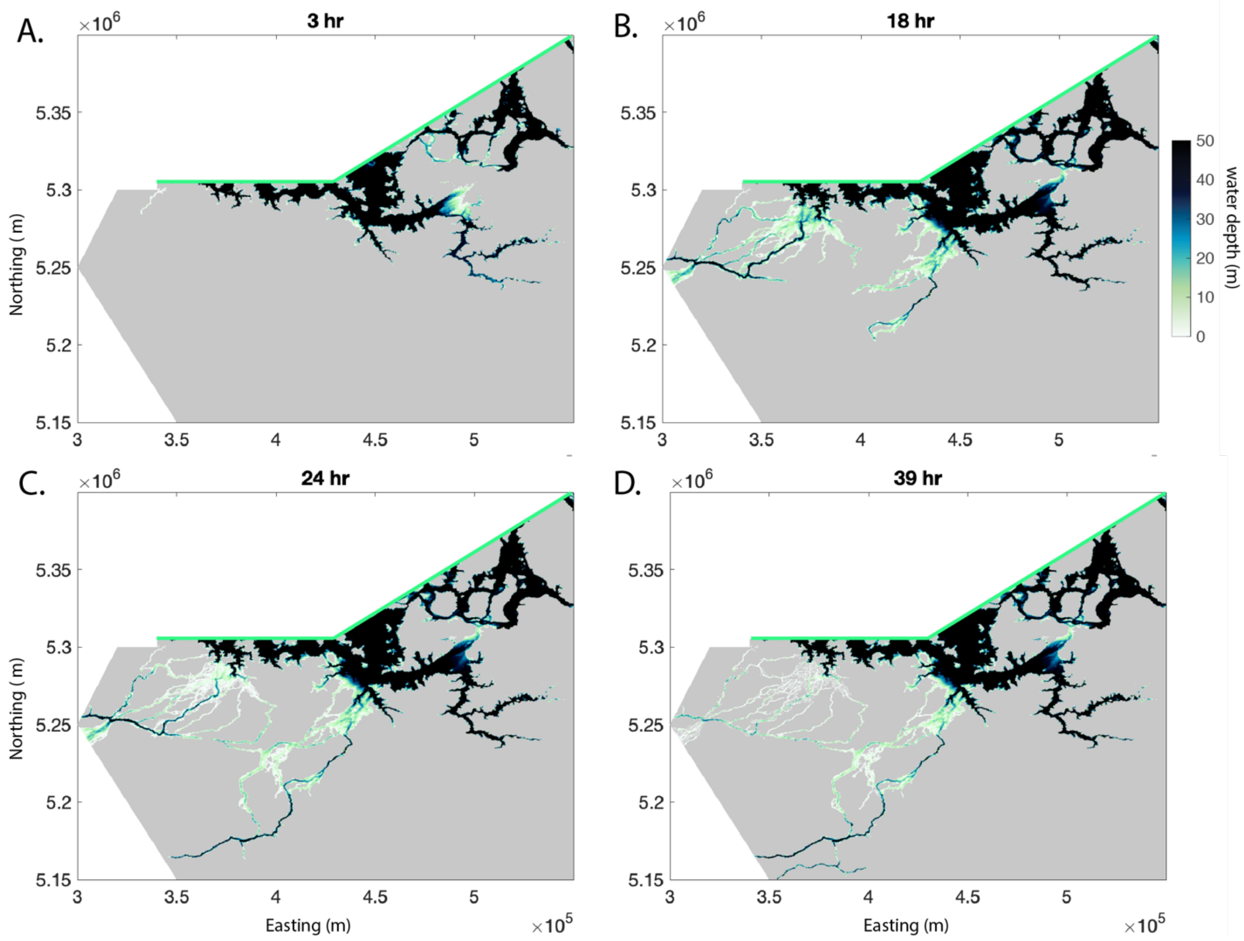

Supplementary Figure 14 | Predicted water depths for flooding simulation on 18 ka topography with a peak input discharge of $6x10^6$ m^3/s (21 hr) as in main text at 3 hrs (A), 18 hrs (B), 24 hrs (C), and 39 hrs (D). Green lines show the location of inlet (inflow boundaries).

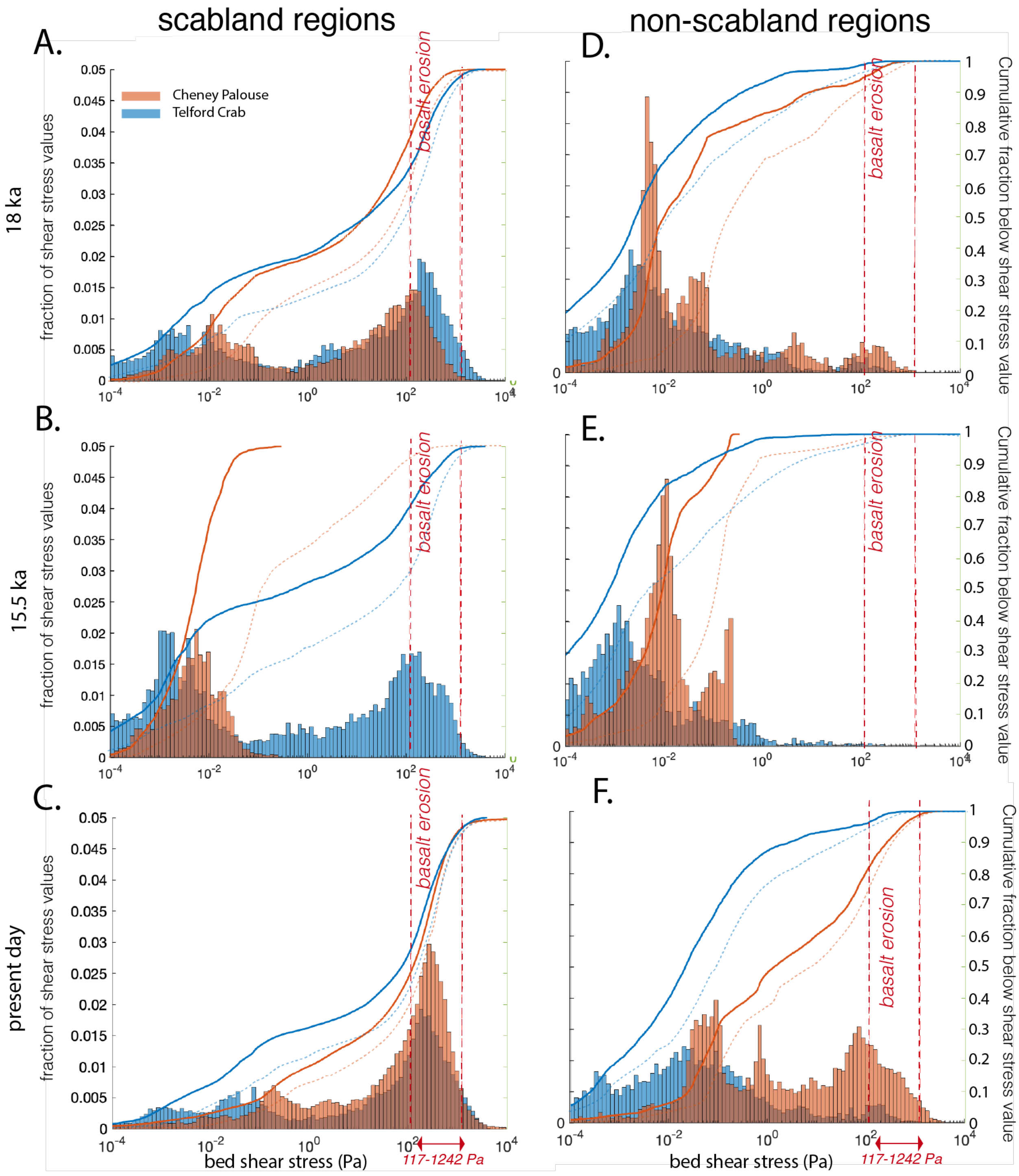

Supplementary Figure 15 | Histograms and empirical cumulative distribution functions for maximum bed shear stresses in Telford-Crab Creek (blue) and Cheney-Palouse (orange) for scabland regions (left side) and non-scabland regions (right side) for $5x10^6$ m^3/s (21 hr) flood event on the 18 ka (A,D), 15.5 ka (B,E), and present-day (C,F) topography. Vertical dashed red lines show threshold shear stress values (117-1242 Pa) required to erode

basalt.

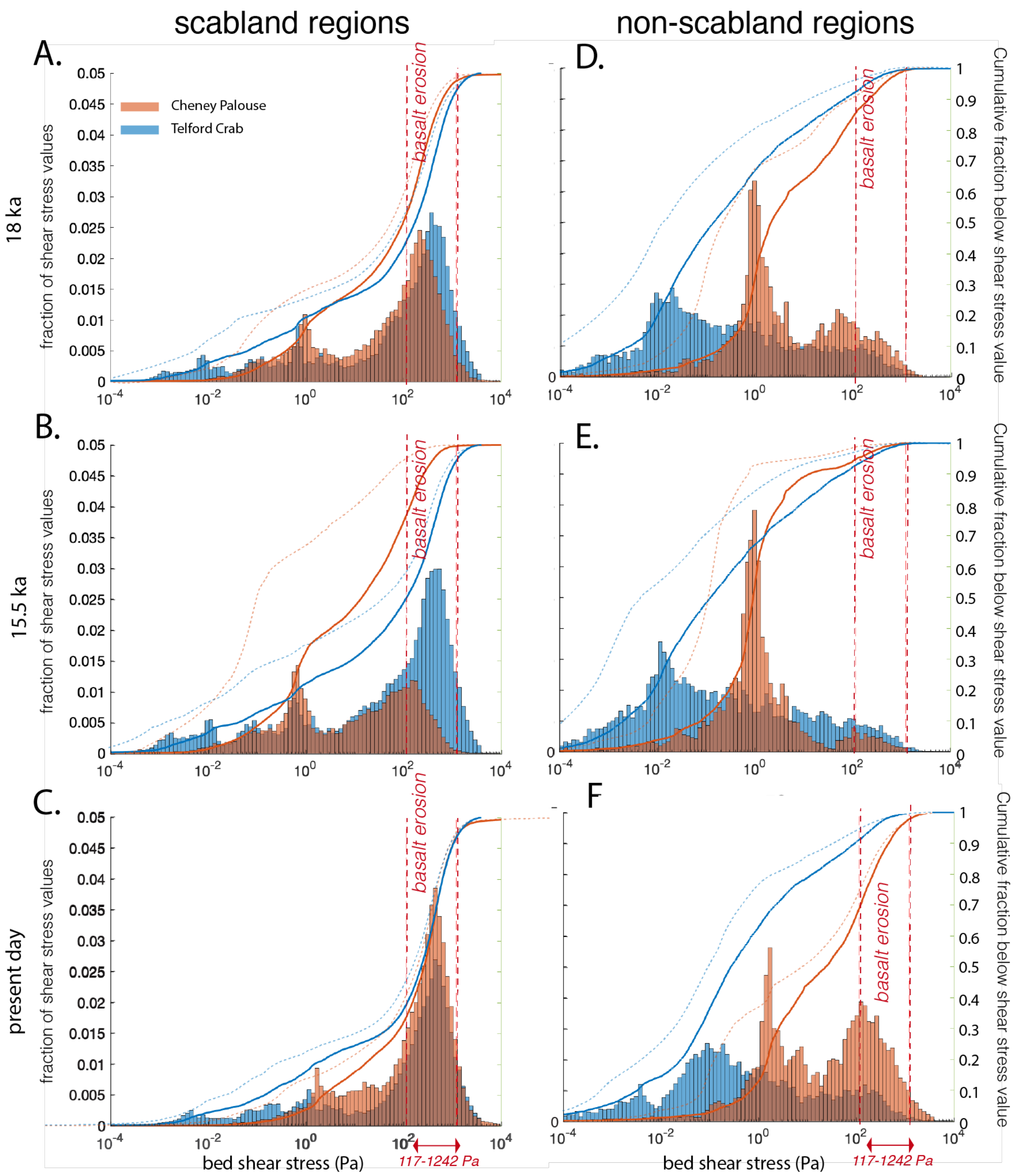

Supplementary Figure 16 | Histograms and empirical cumulative distribution functions for maximum bed shear stresses in Telford-Crab Creek (blue) and Cheney-Palouse (orange) for scabland regions (left side) and non-

scabland regions (right side) for $7x10^6$ m^3/s (21 hr) flood event on the 18 ka (A,D), 15.5 ka (B,E), and present-day (C,F) topography. Vertical dashed red lines show threshold shear stress values (117-1242 Pa) required to erode basalt.

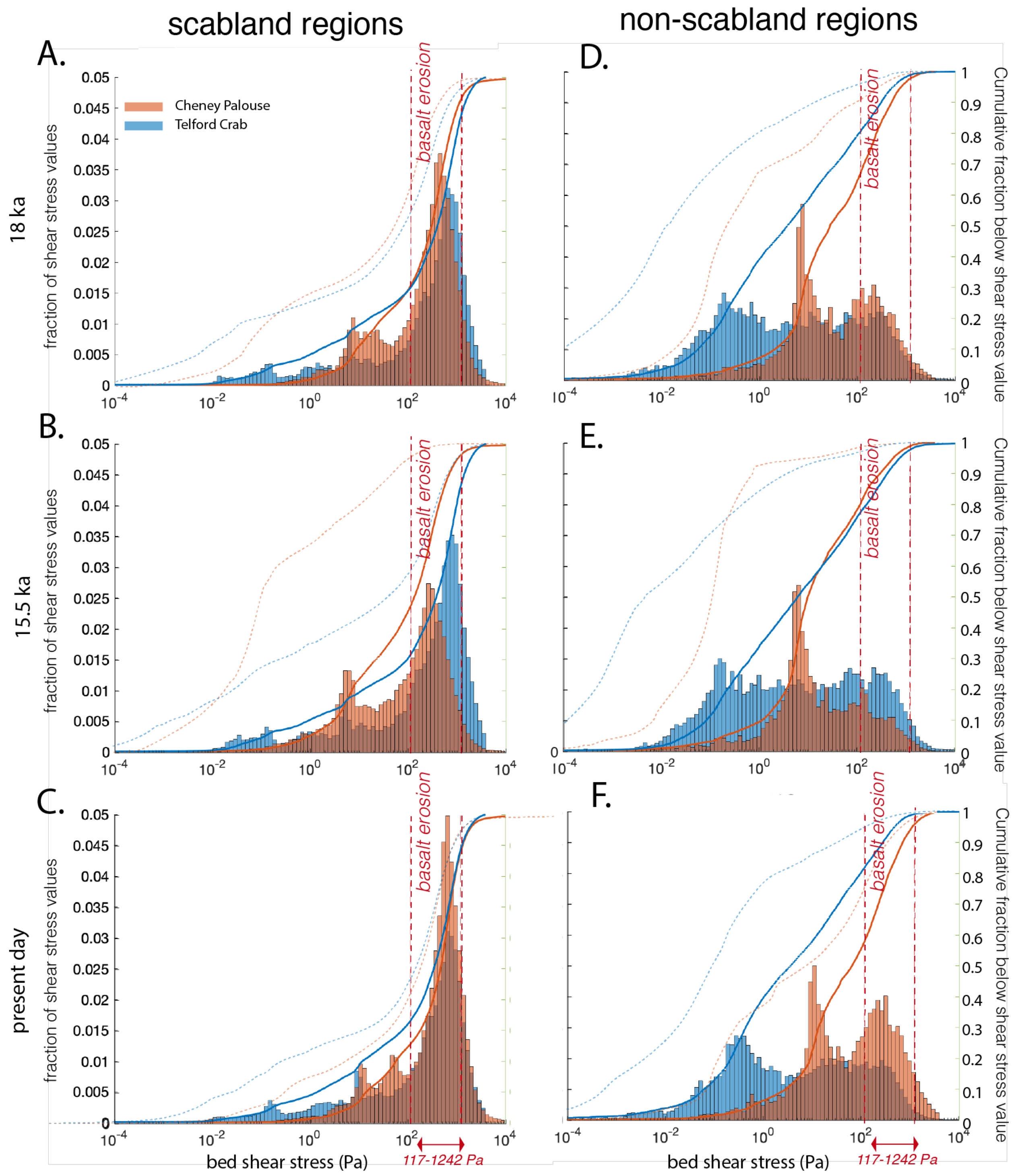

Supplementary Figure 17 | Histograms and empirical cumulative distribution functions for maximum bed shear stresses in Telford-Crab Creek (blue) and Cheney-Palouse (orange) for scabland regions (left side) and non-scabland regions (right side) for 10x10^6 m^3/s (21 hr) flood event on the 18 ka (A,D), 15.5 ka (B,E), and present-day (C,F) topography. Vertical dashed red lines show threshold shear stress values (117-1242 Pa) required to erode

basalt.

REFERENCES

Atwater, B. F. (1987) ‘Status of Glacial Lake Columbia during the Last Floods from Glacial Lake Missoula’, *Quaternary Research*, 201, pp. 182–201.

Baker, V. R. (1978) ‘Paleohydraulics and Hydrodynamics of Scabland Floods’, in *The Channeled Scabland*. NASA, pp. 59–79.

Baker, V. R. *et al.* (2016) ‘Pleistocene megaflood landscapes of the Channeled Scabland’, *Geological Society of America Field Guide*, 0041(01), pp. 1–73. doi: 10.1130/2016.0041(01).

Balbas, A. M. *et al.* (2017) ‘Be dating of late Pleistocene megafloods and Cordilleran Ice Sheet retreat in the northwestern United States’, *Geology*, 45(7), pp. 583–586. doi: 10.1130/G38956.1.

Cosma, T. and Hendy, I. L. (2008) ‘Pleistocene glacimarine sedimentation on the continental slope off Vancouver Island , British Columbia’, *Marine Geology*, 255, pp. 45–54. doi: 10.1016/j.margeo.2008.07.001.

Gombiner, J. H. *et al.* (2016) ‘Isotopic and elemental evidence for Scabland Flood sediments offshore Vancouver Island’, *Quaternary Science Reviews*. Elsevier Ltd, 139, pp. 129–137. doi: 10.1016/j.quascirev.2016.02.026.

Hanson, L. G. (1970) *The Origin and Development of Moses Coulee and other Scabland Features on the Waterville Plateau, Washington*.

Hanson, M. A. and Clague, J. J. (2016) ‘Record of glacial Lake Missoula floods in glacial Lake Columbia , Washington’, *Quaternary Science Reviews*. Elsevier Ltd, 133, pp. 62–76. doi:

10.1016/j.quascirev.2015.12.009.

Lapotre, M. G. A. and Lamb, M. P. (2015) ‘Hydraulics of floods upstream of horseshoe canyons and waterfalls’, *Journal of Geophysical Research : Earth Surface*, pp. 1227–1250. doi: 10.1002/2014JF003412.Received.

Larsen, I. J. and Lamb, M. P. (2016) ‘Progressive incision of the Channeled Scablands by outburst floods’, *Nature*. Nature Publishing Group, 538(7624), pp. 229–232. doi: 10.1038/nature19817.

Lopes, C. and Mix, A. C. (2009) ‘Pleistocene megafloods in the northeast Pacific’, *Geology*, (1), pp. 79–82. doi: 10.1130/G25025A.1.

O’Connor, J. E. *et al.* (2020) ‘The Missoula and Bonneville floods — A review of ice-age megafloods in the Columbia River basin’, *Earth-Science Reviews*. Elsevier B.V., p. 103181. doi: 10.1016/j.earscirev.2020.103181.

Peltier, W. R. (2004) ‘GLOBAL GLACIAL ISOSTASY AND THE SURFACE OF THE ICE-AGE EARTH: The ICE-5G (VM2) Model and GRACE’, *Annual Review of Earth and Planetary Sciences*, 32, pp. 111–149. doi: 10.1146/annurev.earth.32.082503.144359.

Praetorius, S. K. *et al.* (2020) ‘The role of Northeast Pacific meltwater events in deglacial climate change’, *Science Advances*, 6(February), pp. 1–18. doi: 10.1126/sciadv.aay2915.

Schultz, R. A. (1995) ‘Limits on Strength and Deformation Properties of Jointed Basaltic Rock Masses’, *Rock Mechanics and Rock Engineering*, 28, pp. 1–15.

Waitt, R. B. (1985) ‘Case for periodic , colossal jokulhlaups from Pleistocene glacial Lake Missoula’, *Geological Society of America Bulletin*, 96(10), pp. 1271–1286. doi: https://doi.org/10.1130/0016-7606(1985)96<1271:CFPCJF>2.0.CO;2.

Waitt, R. B. (2017) ‘Pleistocene glaciers, lakes, and floods in north-central Washington State’,

Geological Society of America Field Guide, 0049(08), pp. 175–205. doi: 10.1130/2017.0049(08).

Washington State Geologic Survey Surface Geology Map 1:100,000 (2020). Available at: https://www.dnr.wa.gov/programs-and-services/geology/publications-and-data/gis-data-and-databases.
